# Asiatic acid, a novel ciprofloxacin adjuvant inhibits *Shigella flexneri* infection

**DOI:** 10.1080/19490976.2025.2586329

**Published:** 2025-11-30

**Authors:** Priyanka Maitra, Samhati Bhukta, Animesh Gope, Pratanu Kayet, Surajit Basak, Shin-Ichi Miyoshi, Kei Kitahara, Shanta Dutta, Sushmita Bhattacharya

**Affiliations:** a Division of Biochemistry, ICMR-National Institute for Research in Bacterial Infections, Kolkata, India; b Division of Clinical Medicine, ICMR-National Institute for Research in Bacterial Infections, Kolkata, India; c Division of Bioinformatics, ICMR-National Institute for Research in Bacterial Infections, Kolkata, India; d Division of Pharmaceutical Sciences, Graduate School of Medicine, Dentistry and Pharmaceutical Sciences, Okayama University, Okayama, Japan; e Collaborative Research Center of Okayama University for Infectious Diseases in India, ICMR-National Institute for Research in Bacterial Infections, Kolkata, India; f Department of Bacteriology, ICMR-National Institute for Research in Bacterial Infections, Kolkata, India

**Keywords:** *Shigella flexneri*, asiatic acid, ciprofloxacin, adjuvant, membrane damage, depolarization, nuclear damage, efflux inhibitor

## Abstract

Bacterial infection caused by intracellular pathogens such as *Shigella flexneri* is a rapidly increasing global health concern that requires urgent and necessary action. The dearth of licensed vaccines against shigellosis and the decline in susceptibility to conventional antibiotics has encouraged the development of new antibiotic principles and drugs. The treatment options are decreasing faster than the discovery rate of new antibacterial agents. Combinatorial approach of antibiotics with non-antibiotic adjuvants is a promising aspect to treat resistant bacterial infections. Asiatic acid, a membrane-disrupting triterpenoid with wide antimicrobial and immunomodulatory properties, can potentiate antibiotics, but the exact mechanisms remain broadly unexplored. Therefore, in this study, we screened the interaction of asiatic acid with several antibiotics. The results showed synergistic interactions of asiatic acid with antibiotics against susceptible and multidrug-resistant *S. flexneri* clinical isolates. Particularly important was the interaction of asiatic acid with the quinolone antibiotics ciprofloxacin and nalidixic acid. A detailed study showed that combined treatment of asiatic acid with ciprofloxacin inhibited *S. flexneri* biofilm formation and resistance development. An increase in membrane disruption and depolarization upon co-treatment was evident by surface electron and confocal microscopy. In addition, asiatic acid and ciprofloxacin synergism was identified to inhibit efflux activity and intracellular bacterial viability. However, asiatic acid showed no synergistic toxicity with ciprofloxacin towards mammalian cells. The antibacterial activity was further verified in a *S. flexneri* infected mice model. Therapeutic benefits were evident with reduced bacterial burden, recovery from intestinal tissue damage and increase in mice survivability. The results showed that this combination can target the bacterial membrane, efflux pump proteins and biofilm formation, thereby preventing resistance development. The combination treatment offers a proof of concept in targeting essential bacterial activities and might be developed into a novel and efficient treatment alternative against *S. flexneri*.

## GRAPHICAL ABSTRACT

**Figure:**
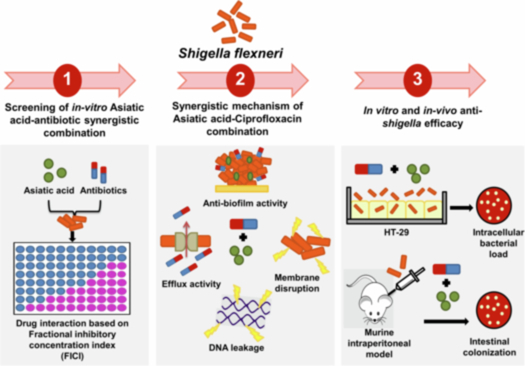

## Introduction

Treatment of bacterial infections is challenging due to the increasing prevalence of antibiotic-resistant pathogens*. Shigella flexneri*, a Gram-negative invasive bacterium associated with bacillary dysentery (bloody diarrhea), was classified as the second major cause of diarrheal death (13.2%) irrespective of age group in 2016, claiming nearly 212,438 lives.^1^ However, infection is more common among children below 5 y and immunocompromised individuals. In 2021, the mortality rate increased from 6.6% (2000) to 8.1%, just beyond the leading factor rotavirus (24.4%) globally among children.^2^ Antibiotic therapy is recommended by the World Health Organization (WHO) to tackle shigellosis.^3^ However, excessive antibiotic usage has led to the rapid emergence of antibiotic resistance among bacterial pathogens.^4^ Currently, meta-analysis data have revealed that nearly 68.7% of strains in Asia to be MDR. Resistance was predominant for WHO recommended 1st choice of drug ciprofloxacin (29.8%), as well as azithromycin (29.2%), and ceftriaxone (23.8%) which are used to treat ciprofloxacin resistant strains.^5^ Even more concerning reports are emerging from India where resistance to ciprofloxacin is as high as 56.2%^6^–83.36%.^7^ Despite these high levels of resistance to ciprofloxacin, it still remains the recommended first line of therapy.^3^ Further, ciprofloxacin resistance is associated with additional resistance to third generation cephalosporins, aminoglycosides, and macrolides, thus creating extensively drug resistant (XDR) strains.^8^ Owing to the high prevalence of fluoroquinolone-resistance, WHO in 2024 has updated *Shigella spp*. as a high-priority pathogen.^9^

In this regard, vaccination could be beneficial for reducing antibiotic usage and thereby resistance. However, multiple serotypes (>50) of the pathogen have led to limited progress in vaccine development. No licensed vaccine against *Shigella* is available in market globally.^10^ Therefore, the WHO has prioritized the development of unconventional or novel interventions for effective treatment and limiting the spread of this high-priority pathogen.^9^

Drug combination is an interesting alternative to address the problem of AMR. The combination of drugs has achieved therapeutic success in treating bacterial infections,^11^HIV,^12^cardiovascular diseases,^13^ and cancer,^14^ with limited knowledge about their mechanism of action. Natural compounds or phytochemicals are often reported as antibiotic potentiators or adjuvants. Several known herbal compounds with their antimicrobial properties possess the unique ability to enhance the efficacy of conventional antibiotics.^15^ In addition, the enhancers showing synergism with antibiotics minimizes side effects and limits resistance development with reduced cost. Hence, these antibiotic potentiators have gained the attention of researchers worldwide and are the need of the hour to tackle AMR.

Asiatic acid (2α, 23-dihydroxyursolic acid), a pentacyclic triterpenoid (PT) extracted from *Centella asiatica*, *Melastoma malabathricum, Shorea robusta,* and other medicinal plants, is well reported to possess anti-inflammatory and antibacterial properties against *Staphylococcus aureus,*^16^ *Mycobacterium tuberculosis,*^17^ *Escherichia coli*,^18^^,^^19^ *Clostridium difficile,*^20^ *Pseudomonas aeruginosa,*^18^^,^^19^ and other bacteria.^21^ Despite its wide range of antibacterial functions, limited knowledge regarding its mechanism of action is available in the literature. Like other terpenoids, it probably acts by disruption of bacterial membrane,^20^preventing biofilm formation,^18^ or bacterial adhesion to host cells.^22^ Besides, we have recently published that asiatic acid intervened host pathogen interaction by inducing antimicrobial peptide gene expression to prevent the intracellular growth of *S. flexneri.*^23^ Apart from its direct antibacterial properties, few studies have shown the synergistic activity of asiatic acid with some antibiotics.^24^^,^^25^ But most of the potentiating activity of asiatic acid with antibiotics is assessed by *in vitro* drug interactions. *In vivo* studies are not reported that can guide for trials in humans.

This study therefore emphasizes to screen the potential synergistic combination of asiatic acid with conventional antibiotics against *S. flexneri* and understand the mode of action behind this adjuvant potential of asiatic acid*.* We further analyzed the mechanism behind Asiatic acid-mediated enhancement of antibiotic efficacy in both cell line and animal model. Overall, this study highlights an effective therapeutic option with a discrete mechanism, therefore holding promising potential for development as an antibiotic adjuvant for combating antibiotic-resistant *S. flexneri* infection.

## Materials and methods

### Chemical and reagents

Asiatic acid (Aa, #546712), chloramphenicol (Chl, #C0378), ampicillin (Amp, #A9518), ciprofloxacin (Cip, #17850), nalidixic acid (Nal, #N8878), tetracycline (Tet, #T7660), streptomycin (S, #S9137), and azithromycin (Az, #PZ0007) were all purchased from Sigma-Aldrich. Asiatic acid was suspended in DMSO. Tetracycline, ampicillin and streptomycin were suspended in molecular grade water. Ciprofloxacin was suspended in 0.1 N HCl, nalidixic acid in 0.5 M NaOH, and azithromycin in 95% ethanol. Respective diluents were used as negative control for all experiments.

### Bacterial strains

*S. flexneri* standard strain (Sf2457T) and multidrug resistant (MDR) clinical *S. flexneri* isolates BCH12654, IDH10994, IDH14386, IDH10932, IDH14363, IDH14351, and IDH14377 were used for this study. All strains used belong to the most prevalent serotype 2a. Strain Sf2457T was obtained from Dr. Hemanta Koley, ICMR NIRBI Kolkata. Clinical isolates were obtained from Dr. Asish K Mukhopadhyay's laboratory, ICMR NIRBI, Kolkata. The resistance profiles of the MDR isolates are provided in Supplementary Table S1.

Bacterial strains were routinely maintained in Mueller Hinton agar (#M173) supplemented with 0.025% Congo red (#GRM927) and cultured in Mueller Hinton broth (#M391) at 37 °C with aeration.

### Cell line and growth conditions

HT-29, a human colorectal adenocarcinoma cell line (#HTB-38), was purchased from the American Type Culture Collection (ATCC). The cells were regularly cultured in complete McCoy's 5A media (#M4892) containing 10% fetal bovine serum (#10082147), 1% nonessential amino acids (#11140050), and 1% penicillin‒streptomycin (#15140122) in a humidified 5% CO_2_ incubator at 37 °C.

### Antimicrobial susceptibility testing

Antibacterial activities of asiatic acid and antibiotics against *S. flexneri* strains were examined using micro broth dilution assay in sterile 96-well polystyrene plate (Corning, NY, USA) following the Clinical and Laboratory Standards Institute recommended guidelines.^26^ Serial dilutions (two-fold) of antibiotics and asiatic acid (Aa) were carried out in cation-adjusted Mueller‒Hinton broth (CA-MHB). The bacterial culture inoculated from the Congo red-positive colony was adjusted to McFarland standards of 0.5 and added to individual wells to achieve an inoculum load of 5 × 10^5^ CFU mL^−1^.^27^^,^^28^ Wells without drug or bacterial inoculum were used as growth and sterility controls, respectively. The plates were kept at 37 °C for 24 h. After incubation, 10 µl of resazurin (0.015%) was added to the wells, and the change in dye coloration was monitored. The minimum dose of a compound that failed to change coloration from blue (resazurin) to pink (resorufin product) was taken as the minimum inhibitory concentration (MIC).^29^

### Drug interaction

The interactions between asiatic acid and different antibiotics against the selected bacterial strains were examined using Checkerboard broth dilution method. Briefly, dilutions of asiatic acid and antibiotics were made based on the MIC value of individual compound in CA-MHB, followed by the addition of bacterial inoculum (5 × 10^5^ CFU mL^−1^). The MIC of the compounds in combination was determined as described above. The interaction between individual asiatic acid–antibiotic combinations was interpreted based on the FICI score via the following formula:

FICI = (asiatic acid MIC in combination)/(asiatic acid MIC alone) + (antibiotic MIC in combination)/(antibiotic MIC alone).

The antibiotics used were ampicillin, chloramphenicol, ciprofloxacin, nalidixic acid, tetracycline, streptomycin, and azithromycin. The interaction between the combinations is depicted by the Fractional Inhibitory Concentration Index (FICI) score and isobologram as prepared with concentrations of asiatic acid (x-axis) and antibiotic (y-axis) from the Checkerboard assay. Briefly, a FICI value of ≤0.5 and concave curve represent synergistic interactions. The FICI scores of >0.5 and <2 and straight line depict additive interactions, whereas the antagonistic interaction is denoted by FICI ≥ 2 and has convex curve.^30^

### Time kill assay

Time- and concentration-dependent antibacterial effects of mono- or combination treatment with asiatic acid and ciprofloxacin against *S. flexneri* growth was investigated. Fresh CA-MHB tubes were inoculated with 5 × 10^5^ CFU mL^−1^ of bacteria.^27^ Cultures were treated with varying concentrations of asiatic acid (156.25−5000 µg mL^−1^) or ciprofloxacin (0.0025−0.04 or 1−16 µg mL^−1^).

To examine synergistic growth inhibition, cultures were treated with asiatic acid (156.25 µg mL^−1^), ciprofloxacin (0.0025 or 2 µg mL^−1^) alone, or in combination with different bacterial strains. Cultures with the respective vehicle control and no antibacterial agent acted as growth control. All the tubes were incubated at 37 °C with aeration. Small volumes of culture media were withdrawn at designated time points from each tube. Ten-fold serial dilution was performed, plated, and kept overnight at 37 °C to determine bacterial viability, which was represented as log_10_ CFU mL^−1^. A log_10_ CFU mL^−1^ reduction of ≥2, between co-therapy and the most effective monotherapy agent alone at 24 h, was considered as synergistic growth inhibition.^31^

### Resistance development assay

Resistance emergence in mono- or combined treatment with sub-MIC concentration of ciprofloxacin and asiatic acid was checked using a previously described protocol.^32^ Briefly, an exponential-phase culture was diluted in CA-MHB supplemented with ciprofloxacin in the absence or presence of asiatic acid and kept at 37 °C for 24 h. The bacterial MIC against ciprofloxacin was checked as described above. Next, these bacterial cultures were treated with sub-MIC of antimicrobial agents for the upcoming passage and MIC was again evaluated. Sequential passaging was performed continuously for 20 d, and the fold change in MIC of ciprofloxacin was plotted and represented graphically.

### Antibiofilm assay

Biofilm inhibition activity was assessed in a flat-bottomed 96-well polystyrene plate using established methodology with minor changes.^33^ Briefly, bacterial culture (10^8^ CFU mL^−1^) supplemented with 1.2% bile salts (#B8756) in Tryptic Soy Broth (#22092) were treated with asiatic acid (1250−39.06 µg mL^−1^) and ciprofloxacin (0.02−0.000625 or 8−0.25 µg mL^−1^) alone or in combination at 37 °C without aeration for 24 h. Sodium dodecyl sulfate (#L3771) was used as a positive control. Bacteria inoculated without bile salt served as a blank. For quantification of biofilm formation, biofilms were washed in PBS and incubated with crystal violet (0.5%) for 10 min. After staining, the biofilms were washed again and air dried. Finally, the stained biofilms were solubilized using ethanol (95%), and the absorbance at 540 nm was measured using microplate ELISA reader (Bio-Tek EL800) and graphically represented as percent biofilm to control.

For quantification of biofilm viability, biofilms were washed and suspended in PBS. Ten-fold serial dilutions were plated in MHA and kept overnight to determine the CFU count.

### Biofilm eDNA extraction and visualization

Extracellular DNA (eDNA) from biofilm matrix was isolated using established methodology with minor modifications.^34^^,^^35^ After removing the planktonic cells, the biofilms were washed with PBS. The biofilm matrix or extracellular polymeric substances (EPS) was then extracted using 1.5 N NaCl and centrifuged at 5000 × *g* for 10 min. The eDNA present in the supernatant (EPS fraction) was measured with NanoDrop (Nabi). Finally, the eDNA was concentrated using ethanol precipitation and visualized on agarose gel.

For *in situ* visualization of the eDNA content of the biofilm matrix, cells grown on coverslips were washed with PBS and stained with TOTO-1 (#T3600) dye at 1:1000 dilution and visualized using CLSM (Zeiss LSM 710).^36^^,^^37^

### Exopolysaccharide visualization and quantification

For visualization of exopolysaccharide, biofilm grown on coverslips were washed with PBS and stained with 50 µg mL^−1^ of FITC-conjugated Concanavalin A (#C7642). Simultaneously, biofilm were co-stained with Concanavalin A and DAPI (2 µg mL^−1^) to quantify EPS and bacterial composition. Z-stack images of biofilms were captured using CLSM (Zeiss LSM 710).

The polysaccharide content of EPS fraction was determined using phenol sulfuric acid method. After treatment, the biofilm matrix was isolated as described above. Then, an equal volume (20 µl) of EPS fraction was mixed with phenol (5%). Finally, 100 µl of sulfuric acid was added, and kept for 10 min at 25 °C . The absorbance was measured at 490 nm and graphically represented.^35^

### Bacterial viability assay

The viability of treated bacterial samples was analyzed using Live/Dead BacLight viability kit (#L7012) as described previously with slight modifications.^38^ Bacterial cultures (10^8^ CFU mL^−1^) were treated with asiatic acid (156.25 µg mL^−1^), ciprofloxacin (0.0025 or 2 µg mL^−1^) alone, or in combination at 37 °C with aeration. After incubation for overnight, the bacterial suspensions were pelleted down at 6000 rpm for 5 min, rinsed with 0.85% NaCl and stained with a 1:1 (SYTO9:propidium iodide) ratio of Live/Dead BacLight reagent in 0.85% NaCl. The samples were stained in the dark for 15 min. Fluorescent bacterial samples were imaged using Confocal Microscope (Zeiss LSM 710).

### Microscopic imaging to detect membrane permeability

The membrane permeability of the treated bacterial samples was analyzed using FM4-64/DAPI staining (#T13320, #D1306) as described previously with slight modifications.^20^^,^^38^ Bacterial cultures (10^8^ CFU mL^−1^) were treated with asiatic acid (156.25 µg mL^−1^) or ciprofloxacin (0.0025 or 2 µg mL^−1^) alone or in combination. After overnight incubation, the bacterial suspensions were pelleted down at 6000 rpm for 5 min and washed with PBS. The cells were subsequently incubated with FM4-64 (1 μg mL^−1^) in the dark for 15  min. Next, the samples were washed and counterstained with DAPI (2  μg mL^−1^) for the same duration in the dark. Fluorescent bacterial samples were imaged using Confocal Microscope (Zeiss LSM 710).

### Surface ultra-structure of bacteria

Morphological changes in the bacterial samples under treatment were imaged using Field emission scanning electron microscopy. The bacterial samples were treated with Asiatic acid or ciprofloxacin alone or in combination. After treatment, the samples were incubated overnight at 4 °C in fixative buffer containing glutaraldehyde (2.5%) and paraformaldehyde (4%). After fixation, the samples were crosslinked for 1 h in osmium tetroxide (1%). In the following step, the samples were subjected to gradual dehydration using ethanol (30%, 50%, 70%, 90%, and 100%) and dried.^20^ Finally, the samples were coated with gold and visualized via a Jeol JSM 7500F SEM with an accelerating voltage of 5 kV.

### Determination of nucleic acid leakage

The bacterial cultures were treated as discussed above and harvested at 10,000 × g for 10 min. Nucleic acid leakage of treated bacterial samples was examined following a previously established protocol.^20^ The clear supernatant obtained was carefully removed. The absorbance was detected at 260 nm for quantification of the nucleic acid. Total nucleic acid was concentrated using ethanol precipitation method and observed via agarose gel electrophoresis.

Simultaneously, treated and untreated cells were stained with TOTO-1 (1:1000 dilutions) as described previously to visualize released eDNA.

### Membrane depolarization

The membrane potential of bacterial samples was analyzed using DiBAC4(3) (#D8189) staining as reported previously with minor modifications.^39^ Cultures were grown to an OD_600_ 0.5 and treated with antimicrobial agents at 37 °C for 4 h with aeration. The cells were then harvested at 6000 × *g* for 5  min, washed in PBS and incubated with DiBAC4(3) (1 µg mL^−1^) for 15  min in the dark. Fluorescent bacterial samples were imaged using Confocal Microscope (Zeiss LSM 710).

### Inner membrane integrity

Exponential bacterial cultures at OD_600_ 0.5 were washed and treated with asiatic acid (156.25 µg mL^−1^) or ciprofloxacin (0.0025 or 2 µg mL^−1^) alone or in combination for 4 h. The treated samples were washed in PBS, mixed with 10 nM propidium iodide (PI; #P4864) and kept in the dark for 30 min. The fluorescence intensity of PI was determined using spectrofluorometer (BMG Labtech) at 535 nm (excitation) and 615 nm (emission) wavelengths.^40^

### Immunoblotting

Asiatic acid- and ciprofloxacin-treated and co-treated bacterial cultures were washed in chilled PBS and ruptured using bacterial lysis buffer supplemented with protease inhibitor and PMSF. The total bacterial protein fraction was harvested by centrifugation at 4 °C for 20 min at 7000 × *g*. In the next step, the protein level was estimated by BCA assay kit (#23225), processed and run on 10% SDS‒PAGE gel. After transferring and blocking (5% nonfat milk, 1 h), the membranes were incubated overnight with an anti-GAPDH antibody (#D16H11) (1:3000), anti-AcrA antibody (#BB-SAP50) (1:3000), and anti-AcrB antibody (#BB-SAP50) (1:3000) at 4 °C. The following day, the membranes were incubated with secondary antibody (1:10,000) and washed with TBST. The bands were finally imaged using HRP substrate (Millipore) in a ChemiDoc MP Imaging System (Bio-Rad, USA). The relative fold change in protein expression was determined after normalization against GAPDH^41^^,^^42^ using Image Lab software (version 5.2.1).

### Quantitative PCR

Bacterial subcultures grown to OD_600_ of 0.5 were harvested for RNA isolation using TRIzol reagent (Invitrogen). DNA contamination was removed using DNA-free kit (Ambion). cDNA was synthesized using cDNA synthesis kit (Thermo Scientific) following the manufacturer's protocol. qRT‒PCR was run on ABI 7500 Fast real-time PCR system using SYBR Green master mix kit (#A25742). The expression levels were normalized using 16 s rRNA (internal control), and the fold change was calculated using 2^−ΔΔC^ method. The primer sequences used are listed in Supplementary Table S4.

### Molecular docking

The 3D structures of AcrA and AcrB of Gram-negative bacteria were obtained from PDB database, and the structures of asiatic acid, ciprofloxacin, and PAβN (MC-207110) were obtained from the PubChem database. Molecular docking was executed using Autodock vina software,^43^ and the probable docking position of AcrA and AcrB was taken from the references.^44–46^ After collecting the probable positions, we first docked the antibiotic (ciprofloxacin) at the substrate binding site of AcrA and AcrB. The docked complex of AcrA and AcrB with ciprofloxacin was then used to dock PAβN and asiatic acid. Finally, Discovery Studio visualizer^47^ was used to collect the bonding information between AcrA and AcrB with above mentioned compounds in their best docked position.

### EtBr efflux kinetics

Ethidium bromide efflux assays were performed using previously established protocol.^48^ Briefly, *S. flexneri* cultures (Sf2457T/BCH12654) were grown to the exponential phase (O.D_600 _ of 0.6). The cultures were then loaded with 20 µg mL^−1^ of EtBr and incubated for 1 h at RT. The bacterial cultures were then diluted in PBS to attain an OD_600_ of 0.2. The cells were then rinsed and resuspended in PBS containing 0.4% glucose and treated with asiatic acid (625−78.125 µg mL^−1^). PAβN (#P4157) was used as a positive control. Fluorescence was detected using fluorescence spectrophotometer (BMG Labtech) at excitation and emission wavelengths of 520 and 590 nm, respectively, for 1 h at 5 min intervals.

### Cytotoxicity assay

To determine the toxicity of asiatic acid and antibiotic treatment in HT29 cells, an MTT (#CT02) dye assay was performed.^49^ Shortly, cells (1 × 10^4^) were cultured in plate (96 well) and kept for 24 h in 5% CO_2_ incubator at 37 °C. The following day, the cells were subjected to different doses of asiatic acid, antibiotics alone, or in combination, keeping respective vehicle as a control for 24 h. After treatment, MTT solution (20 µl of 5 mg mL^−1^) was added to the wells, and the mixture was further kept for 4 h. The formazan crystals that precipitated were dissolved using DMSO. The color intensity was measured using microplate ELISA reader (Bio-Tek EL800) at 570 nm and graphically represented as the percent viability normalized to the control.

### Infection assay

The invasion assay was performed using protocol described earlier.^50^ In brief, 1 × 10^5^ HT29 cells were cultured in plates (6 wells) and incubated in a 5% CO_2_ incubator at 37 °C to reach confluency in monolayer. Subsequently, an overnight bacterial culture from a single colony was subcultured to an OD_600_ 0.5. Thereafter, the bacterial cultures were harvested at 6000 × *g* for 5 min, washed in sterile PBS and dissolved in culture media lacking antibiotics. Infection was performed at a 100:1 multiplicity of infection (MOI). The plates were centrifuged at 700 × *g* for 15 min and kept for 2 h to facilitate bacterial invasion. Following infection, residual bacteria were removed by washing with PBS and incubated with gentamicin solution (50 µg mL^−1^) in culture media for 2 h. Next, the infected cells were again washed, and based on the bacterial strain subjected to different doses of asiatic acid, antibiotics treatment alone, or in combination for 24 h in culture media containing 10 µg mL^−1^ of gentamicin. Based on asiatic acid and/or antibiotic treatment, respective vehicle or vehicle combination was used as a negative control. Post-treatment, the cells were thoroughly washed in PBS and lysed with 0.1% Triton X-100 at 37 °C for 5 min. Ten-fold dilutions of the lysed suspensions were spread onto MHA and kept overnight at 37 °C to determine the % intracellular viability from the CFU count.

### Animals

Adult BALB/c mice approximately 20–24 g in weight were used for the animal experiments. The mice were collected from ICMR-NIRBI, Kolkata, Animal Resources Department. The animals were kept under recommended laboratory conditions with 24 °C temperature and 75% humidity condition in a separate animal house. The animals were given a quality pellet diet and sufficient water.

### *In-vivo* treatment

Adult BALB/c mice were taken as a model for *in vivo* experiments. Food was withdrawn from all the cages 24 h prior to infection. The fasted mice were unbiasedly divided into groups (*n* = 6/group) and intraperitoneally infected with 10^8^ CFU of *S. flexneri* (Sf2457T/BCH12654).^51^ 4 h post infection, the mice were orally administered with vehicle, asiatic acid (25 mg kg^−1^),^52^^,^^53^ ciprofloxacin (1 or 20 mg kg^−1^)^54^^,^^55^ alone, or in combination. The control group, without infection, was also orally subjected to vehicle in the same manner.

Similarly, infected mice were treated with nalidixic acid (5 or 10 mg kg^−1^) alone,^56^ in combination with asiatic acid (25 mg kg^−1^).

### Survival Kinetics

The mortality of mice subjected to intraperitoneal infection with *S. flexneri* (Sf2457T/BCH12654), followed by treatment with ciprofloxacin (1 or 20 mg kg^−1^) in the presence or absence of asiatic acid (25 mg kg^−1^), was monitored over the next 3 d. The survival kinetics of the mice were plotted using Kaplan–Meier analysis curve.

### *In vivo* drug efficacy

The mice were subjected to infection and treatment, similar to the *in vivo* survival kinetics study. Sixteen hours post-infection, blood samples were collected from the mice. The animals were then euthanized, and the colon, spleen, and liver were collected aseptically. The colonic tissues were transversely cut open and washed to remove the luminal contents. Next, all the tissue samples were treated with gentamicin solution (100 µg mL^−1^) for 1 h. After gentamicin treatment, the tissues were washed in PBS, homogenized, ten-fold serially diluted in sterile PBS and spread plated on MHA supplemented with Congo red dye to determine the bacterial load.

### Immunohistochemistry

Formalin-fixed colonic tissue sections were deparaffinized, serially rehydrated, and antigen retrieval was carried out in sodium citrate buffer (pH 6.0). The tissues were permeabilized (0.1% Triton X, 10 min), washed, and blocked in blocking solution (3% bovine serum albumin and 5% FBS in PBS) for 1 h. Next, the tissues were incubated with an anti-*Shigella* antibody (#ab65282) at a 1:100 dilution for overnight at 4 °C. The tissues were further washed with PBST (1× PBS containing 0.1% Tween 20) and incubated with TRITC-conjugated anti-rabbit secondary antibody (#T6778) at a 1:500 dilution for 2 h. After being washed with PBST, the tissues were stained with DAPI and mounted using ProLong Diamond Antifade media and visualized using Axio Observer (Zeiss).

### Histopathological study

Treated mice tissues were fixed with buffered formalin (10%), followed by gradual dehydration with ethanol (50%–100%) and xylene. The tissues were then carefully embedded in paraffin solution (56 °C–58 °C) at 58 ± 1 °C and incubated for 4 h. Following deparaffinization with xylene, the tissues were stained using hematoxylin and eosin (H&E) and mounted on a clean cover slip using DPX. Images were captured using a bright field microscope (Motic, Germany) to visualize histological variations among the control, infected and treatment groups. Colonic sections were analyzed for epithelial fenestration using ProgRes CapturePro software, and % Fenestration [(length of the colonic epithelium section with signs of fenestration/total length of the colonic epithelium section)*100] was calculated.^57^

### Statistical test

The results obtained in this study were statistically compared using GraphPad Prism 8 software (San Diego, USA). The results are represented as mean ±SEM of three individual biological replicates, and statistical comparisons among groups were determined using the most significant statistical test (*t*-test, one-way ANOVA, or two-way ANOVA). The results were regarded as statistically significant at *p* < 0.05. Differences among groups are represented in the figures by distinct superscript letters.

## Results

### Combination of asiatic acid with common antibiotics exhibited synergism

Previously, we have shown that asiatic acid, a triterpenoid, ameliorates *S. flexneri* intracellular growth by activating antimicrobial peptide gene (AMP) expression in intestinal cells.^23^ Herein, we investigated the interaction of asiatic acid with different antibiotics. The antibiotic panel included a cell wall synthesis inhibitor (ampicillin), protein synthesis inhibitors (streptomycin, tetracycline, chloramphenicol, and azithromycin) and DNA synthesis inhibitors (nalidixic acid and ciprofloxacin). An antimicrobial susceptibility assay against the standard *S. flexneri* ATCC strain was performed using asiatic acid–antibiotic combination. Interestingly, the screening results showed that asiatic acid has a synergistic effect and significantly reduces the susceptible dose of ampicillin, chloramphenicol, ciprofloxacin, and nalidixic acid, with FICI score of 0.374, 0.5, 0.25, and 0.375, respectively (Supplementary Table. S2). However, the remaining antibiotics showed additive or antagonistic interaction with asiatic acid. Next, to test the spectrum of asiatic acid as an adjuvant against *S. flexneri* infection, we checked its interaction against a panel of MDR clinical isolates. In line, asiatic acid restored the susceptibility of the majority of the MDR isolates to antibiotics, similar to the standard strain. Overall, potentiating effect was observed for ampicillin (5 strains), chloramphenicol (4 strains), ciprofloxacin (6 strains), and nalidixic acid (5 strains), with FICI value ranging from 0.25 to 0.5, 0.28 to 0.5, 0.25 to 0.5, and 0.25 to 0.5, respectively (Supplementary Table S2). These findings suggest that asiatic acid is a potential broad-spectrum antibiotic adjuvant for combating MDR *S. flexneri* infection.

**Figure 1.**
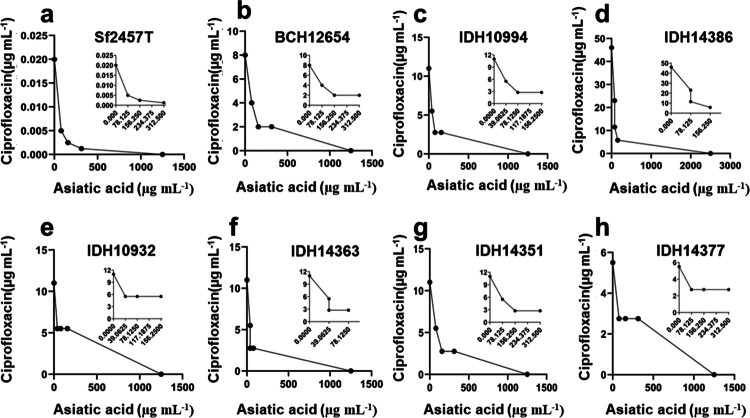
Asiatic acid (Aa) potentiates the activity of antibiotics. Based on the MIC value of asiatic acid and ciprofloxacin, a checkerboard microbroth dilution assay was performed to prepare an isobologram demonstrating the interaction between asiatic acid and ciprofloxacin against *Shigella flexneri* standard and resistant strains. Insets show precise concentrations of asiatic acid (a–h). Data are representation of three independent biological replicates.

Despite the loss of sensitivity towards ciprofloxacin, it still remains WHO recommended first choice of antibiotic for shigellosis.^3^ Due to the clinical and therapeutic importance of ciprofloxacin as an anti-*shigella* drug and the results showing that the Asiatic acid‒ciprofloxacin combination is the most effective synergistic pair, we further focused our study on this combination. The interaction between ciprofloxacin and asiatic acid on the growth of all the bacterial strains studied here was further confirmed by the isobologram method. An isobolograms with a concave curve demonstrated synergistic growth inhibition against standard and most of the clinical isolates **(**Figure 1**)**. These results validate the synergism between asiatic acid and ciprofloxacin against *S. flexneri* infection.

### Asiatic acid prevents ciprofloxacin driven resistance development

**Figure 2.**
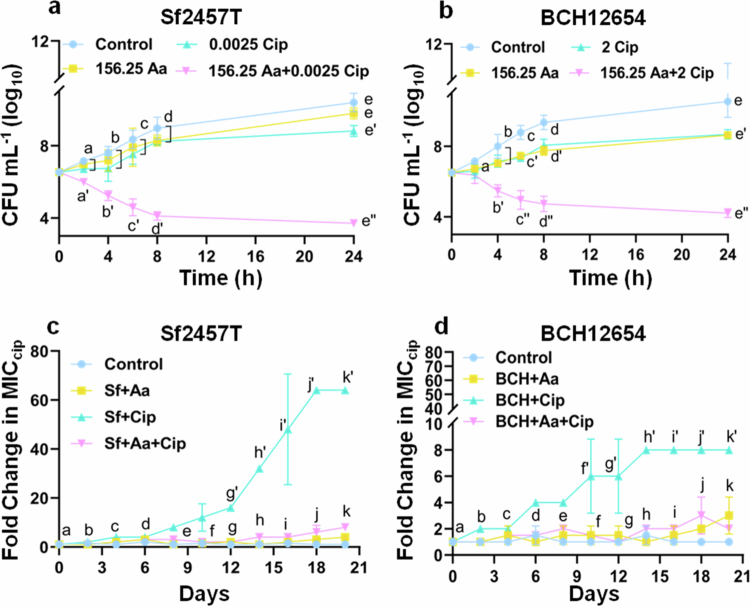
Asiatic acid (Aa) treatment increases ciprofloxacin (Cip) efficacy and hinders the development of resistance. 5 × 10^5^ CFU mL^−1^ of *S. flexneri* cultures were treated with asiatic acid, ciprofloxacin alone, or in combination, and the time-dependent killing curves were assessed by CFU count and plotted as log_10_ CFU mL^−1^ for Sf2457T (a) and the resistant strain BCH12654 (b). The emergence of ciprofloxacin resistance in *S. flexneri* 2457T (c) and BCH12654 (d), during 20 serial passages in sub-MIC concentration of asiatic acid and ciprofloxacin in combination or alone was determined based on an increase in the fold change of MIC compared to the 0th day. The data are presented as mean of three independent biological replicates ±S.E.M. Statistical significance was determined using ANOVA test (two-way). Results are regarded as statistically significant at *p* < 0.05. Differences among groups are represented by distinct superscript letters.

Treatment with asiatic acid and ciprofloxacin monotherapy showed dose dependent *S. flexneri* (Sf2457T and BCH12654) growth inhibition (Supplementary Figure S1). To further investigate the synergistic interaction between ciprofloxacin and asiatic acid, time kill assay was performed. Cotherapy doses showing highest synergistic interaction (lowest FICI score) against Sf2457T [(0.25); 156.25Aa + 0.0025Cip µg mL^−1^] and BCH12564 [(0.375); (156.25Aa + 2Cip µg mL^−1^)] **(**Figure 1) were selected to investigate the underlying mechanism of synergism against planktonic growth. The combination treatment of standard *S. flexneri* strain showed 1.5, 4, and 5 log reduction in bacterial CFU at 4, 8, and 24 h, respectively, compared to ciprofloxacin alone (Figure 2a). Similarly, combination therapy led to 1.5, 3, and 4 log reductions in CFU of *S. flexneri* clinical isolate (BCH12654) at 4, 8, and 24 h, respectively (Figure 2b). Henceforth, ≥2 log reduction in the viability of both bacterial strains was observed at 24 h, demonstrating the synergistic growth inhibitory effect in the presence of asiatic acid and ciprofloxacin combination (Figure 2a,b).

Antibiotics exert selective pressure for the development of drug resistance in bacteria. Hence, we checked the preventive role of asiatic acid against ciprofloxacin driven resistance development. *S. flexneri* was serially passaged with sub-MIC doses of combination or individual antimicrobial agent and the change in ciprofloxacin MIC was monitored for the next 20 d.

Interestingly, the results showed that treatment of asiatic acid along with ciprofloxacin could resist the selection pressure, thereby preventing significant increase in MIC of ciprofloxacin for both the strains. Whereas, ciprofloxacin treatment alone resulted in the development of resistance, with an increase in MIC by 64 and 8 fold for standard and MDR strain respectively (Figure 2c,d). These results suggest that asiatic acid restricts the development of ciprofloxacin resistance in *S. flexneri*.

### Asiatic acid mono and combined therapy with ciprofloxacin prevents biofilm formation in *S. flexneri*

Biofilm formation facilitates the virulence and survivability of *S. flexneri* aiding in cellular invasion.^58^ In addition, biofilm formation also hinders the penetration/uptake of antibiotics. Therefore, a biofilm assay was carried out to check the effects of asiatic acid and ciprofloxacin co-treatment on *S. flexneri* biofilm development. Crystal violet (CV) staining was performed to check biofilm formation and CFU measurement for bacterial biomass assessment.

**Figure 3.**
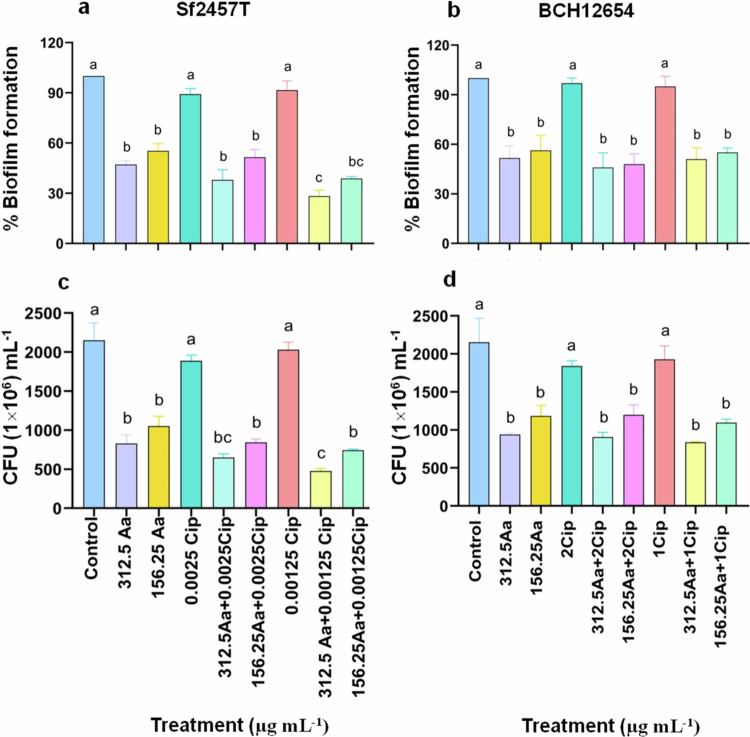
Asiatic acid (Aa) mono- and co-therapy with ciprofloxacin (Cip) inhibited *S. flexneri* biofilm formation. *S. flexneri* (Sf2457T/BCH12654) cultures were treated with asiatic acid (156.25 and 312.5 µg mL^−1^) in the presence or absence of ciprofloxacin (0.00125 and 0.0025 or 1 and 2 µg mL^−1^) for 24 h in biofilm forming conditions (1.2% bile salt). The inhibition of biofilm formation was quantified by measuring O.D. at 540 nm of the crystal violet-stained biofilms and graphically represented as % biofilm formation (a and b), and viability was assessed by plate count method and represented as CFU mL^−1^ (c and d). The data are presented as mean of three independent biological replicates ±S.E.M. Significance was determined using ANOVA test (One way), where *p* < 0.05 was considered statistically different. The distinct superscript letters denote differences among the groups.

Asiatic acid showed more potent antibiofilm activity, preventing biofilm formation as well as bacterial biomass reduction of both the strains compared to ciprofloxacin (Supplementary Figures S2 and S3). Additionally, significant lowering of biofilm formation and biofilm viability was observed in both *S. flexneri* standard and BCH12654 clinical strains treated with asiatic acid and asiatic acid plus ciprofloxacin, as compared to ciprofloxacin monotherapy (Figure 3a–d). However, compared to asiatic acid monotherapy, only 312.5Aa + 0.00125Cip co-treatment group significantly reduced biofilm formation and viability in the standard strain (Figure 3a,c).

**Figure 4.**
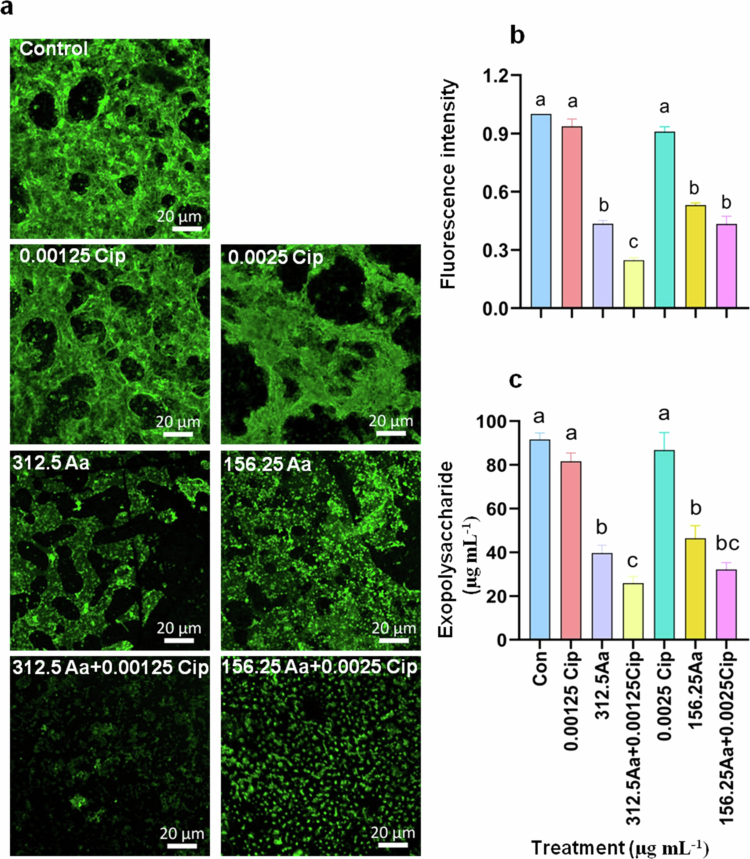
Biofilm matrix (EPS) formation of *S. flexneri* is disintegrated in the presence of asiatic acid (Aa) and the combination treatment. *S. flexneri* (Sf2457T) cultures were grown in the presence of asiatic acid (156.25–312.5 µg mL^−1^), ciprofloxacin (0.0025–0.00125 µg mL^−1^ alone or in combination for 24 h, in biofilm-inducing conditions (1.2% bile salt). The formation of the biofilm matrix was visualized by staining exopolysaccharide with FITC-conjugated Concanavalin A (FITC-ConA) (a), and the mean fluorescence was quantified (b). Changes in exopolysaccharide content, exposed to treatment conditions was determined using Phenol-sulfuric acid method and represented graphically (c). The images are representative of three independent biological replicates, and the data were evaluated from three independent biological replicates and are represented as ±S.E.M. Statistical significance was evaluated by performing One-way ANOVA. The results are statistically significant at *p* < 0.05. Groups statistically different are marked by different superscript letters.

Development of a mature biofilm requires the formation of a matrix that acts as an adhesive molecule to structurally hold the adhered bacteria within the newly formed community. Exopolysaccharide (EPS) is one of the vital matrix components of bacterial biofilms.^36^^,^^59^^,^^60^ Therefore, FITC-Concanavalin A-stained exopolysaccharide was used to investigate the formation of the biofilm matrix. Two doses of cotherapy [Aa + Cip (312.5 + 0.00125) and (156.25 + 0.0025) µg mL^−1^] that prevented biofilm formation (Figure 3) as well as showed synergistic effect against planktonic *S. flexneri* (Sf2457T) culture (Figure 1) were selected. Untreated and ciprofloxacin (0.0025 and 0.00125 µg mL^−1^) treated bacteria showed the presence of densely packed EPS layer (Figure 4a,b). Compared to all the monotherapy groups, significantly loose or structurally disintegrated EPS layer was observed upon co-treatment with 312.5Aa and 0.00125Cip. Asiatic acid (156.25 and 312.5 µg mL^−1^) treatment alone or other combinatorial dose with ciprofloxacin resulted in similar level of EPS disruption compared to untreated control or ciprofloxacin (0.0025 or 0.00125 µg mL^−1^) monotherapy (Figure 4a,b). Simultaneously, quantification of exopolysaccharide level confirmed similar results in line with confocal imaging (Figure 4c).

**Figure 5.**
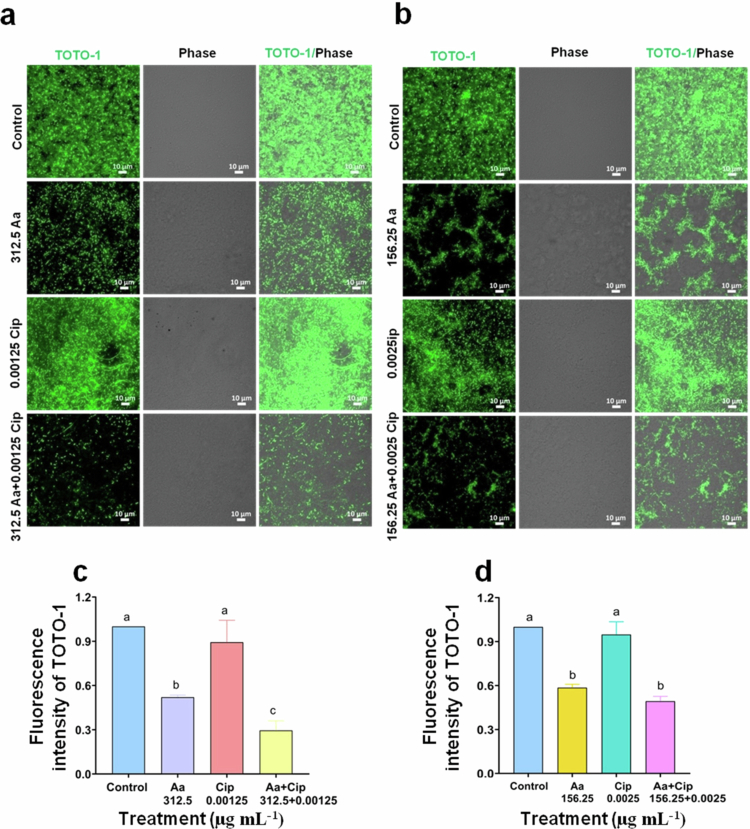
The extracellular DNA (eDNA) content of *S. flexneri* biofilm matrix decreased upon treatment with asiatic acid mono- or co-therapy. *S. flexneri* (Sf2457T) cultures were grown in the presence of asiatic acid (156.25 or 312.5 µg mL^−1^) and ciprofloxacin (0.0025 or 0.00125 µg mL^−1^) individually or in combination [(156.25Aa + 0.0025Cip) and (312.5Aa + 0.00125Cip)] for 24 h in biofilm-inducing conditions (1.2% bile salt). The biofilm was stained with TOTO-1 to visualize eDNA (a and b), and the mean intensity was quantified (c and d). Images are representative of three independent biological replicates, and data are representative of three independent biological replicates represented as ±S.E.M. Statistical significance was evaluated by performing One-way ANOVA (multiple comparisons). The results are statistically significant at *p* < 0.05. Group differences are marked by different superscript letters.

**Figure 6.**
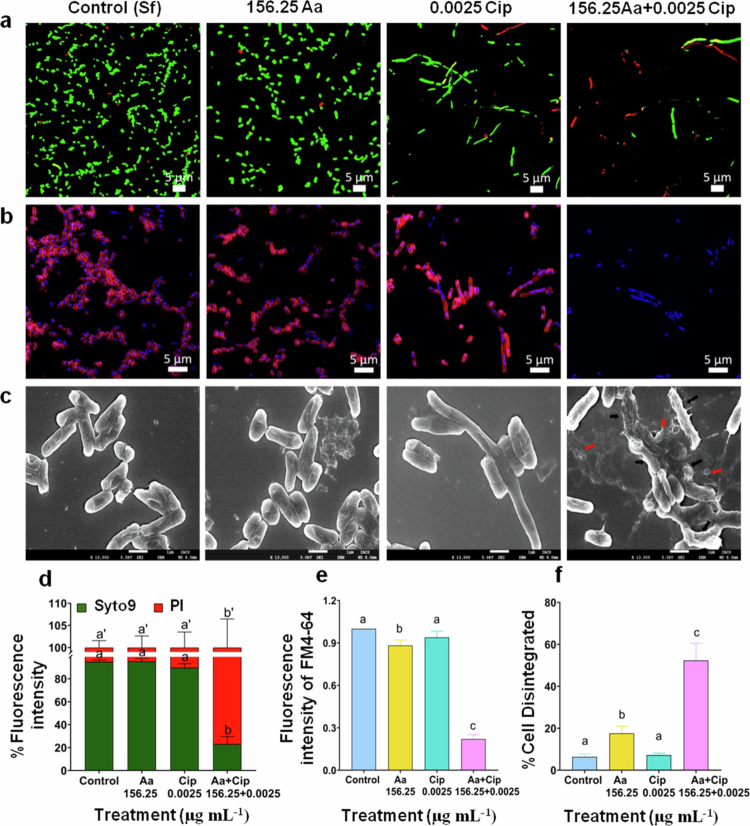
Asiatic acid (Aa) and ciprofloxacin (Cip) co-administration hampers *S. flexneri* membrane integrity. *S. flexneri* (Sf2457T) cultures were treated with mono- or co-therapy of asiatic acid and ciprofloxacin. Confocal microscopy was used to visualize the viability of *S. flexneri* by Syto9/PI staining (a), and the mean intensities were quantified (d). Membrane integrity was assessed by FM4-64/DAPI staining using confocal microscopy (b), and the mean intensities were plotted (e). Surface structural and morphological changes of treated *S. flexneri* were visualized using FE-SEM. The cells with damaged membranes are marked with black arrows, and the red arrow indicates lysed cellular debris (c). The percentage of cell disintegration was calculated after analyzing 100 bacteria per group from SEM data and represented graphically (f). All the images are representative of three independent biological replicates, and the data are represented as mean ±S.E.M. Statistical significance was determined using ANOVA test (one- or two-way). Results are statistically significant at *p* < 0.05. Group differences are marked by distinct superscript letters.

Further, quantification of exopolysaccharide and the bacterial composition of the biofilm (Sf2457T) confirmed a significant reduction in both the parameters, upon co-treatment (312.5Aa and 0.00125Cip) compared to the monotherapy agents (Supplementary Figure S4a,b). Another important component of the biofilm matrix is extracellular DNA (eDNA), majorly contributed by autolysis of cells or secreted by live cells.^36^ The presence of eDNA stabilizes the biofilm, helps to replenish nutritional requirements and contributes to the resistance.^36^^,^^59^^,^^60^ Therefore, we checked the presence of eDNA in the EPS of *S. flexneri* biofilm matrix. The membrane impermeable dye TOTO-1 is specifically used to visualize eDNA. Biofilms were stained with TOTO-1 to visualize the eDNA content of the biofilm matrix. The presence of substantial amount of eDNA was visualized in untreated and ciprofloxacin (0.0025 or 0.00125 µg mL^−1^)-treated bacterial biofilms. Similar to exopolysaccharide, combination treatment with 312.5Aa and 0.00125Cip resulted significant decrease in eDNA compared to monotherapy groups, which was not observed for the other co-treatment group (Figure 5a–d). Similarly, O.D. measurement and agarose gel electrophoresis showed the presence of substantial eDNA in the matrix of untreated and ciprofloxacin (0.00125 µg mL^−1^)-treated bacterial biofilms. Asiatic acid treatment lowered the eDNA level, which was further reduced upon co-treatment (Supplementary Figure S5a,b). Therefore, a reduction of biomass and disruption of a well-developed matrix composed of exopolysaccharide and eDNA might contribute to increased susceptibility of *S. flexneri* biofilm towards ciprofloxacin in the presence of asiatic acid. Taken together, these results enumerate the synergistic potential of asiatic acid as a ciprofloxacin enhancer that mitigates biofilm formation.

**Figure 7.**
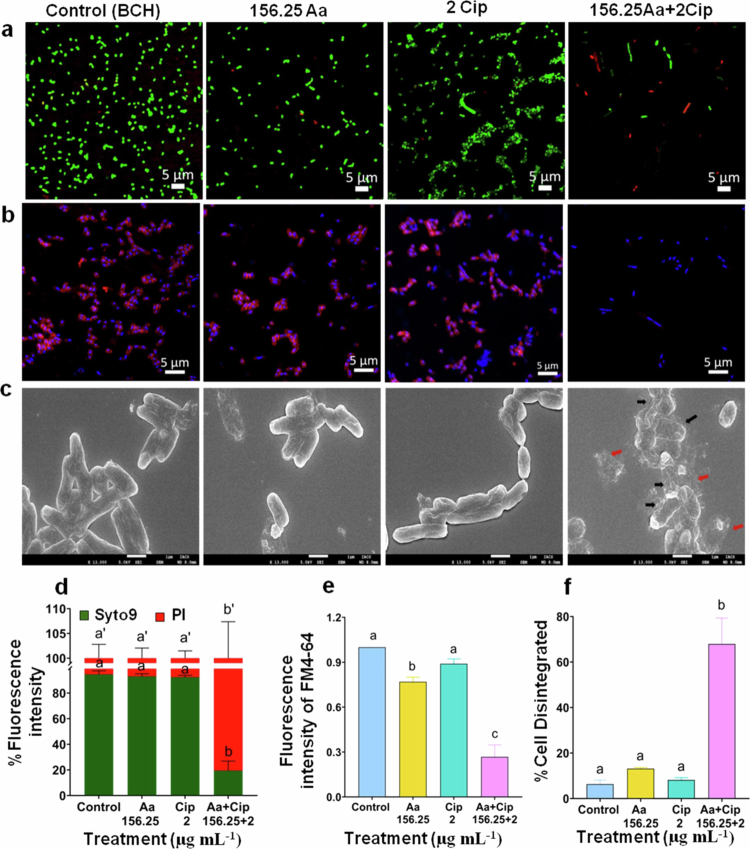
Asiatic acid (Aa) and ciprofloxacin (Cip) in combination disrupt the membrane integrity of *S. flexneri* resistant strain BCH12654. *S. flexneri* (BCH12654) cultures were treated with mono- or co-therapy of asiatic acid and ciprofloxacin. Confocal microscopy was used to visualize the viability of *S. flexneri* by Syto9/PI staining (a), and the mean intensities were quantified (d). Membrane integrity was assessed by FM4-64/DAPI staining (b), and FM4-64 fluorescence was quantified (e). Surface structural and morphological changes of treated *S. flexneri* were visualized using FE-SEM. The cells with damaged membrane are marked with black arrows, and the red arrow marks indicate lysed cellular debris (c). The percentage of cell disintegration was calculated after analyzing 100 bacteria per group from SEM data and represented graphically (f). All images are representative of three independent biological replicates, and the data are represented as mean ±S.E.M. Statistical significance was determined using ANOVA test (one- or two-way). The results are statistically significant at *p*<0.05. Differences among groups are marked by different superscript letters.

**Figure 8.**
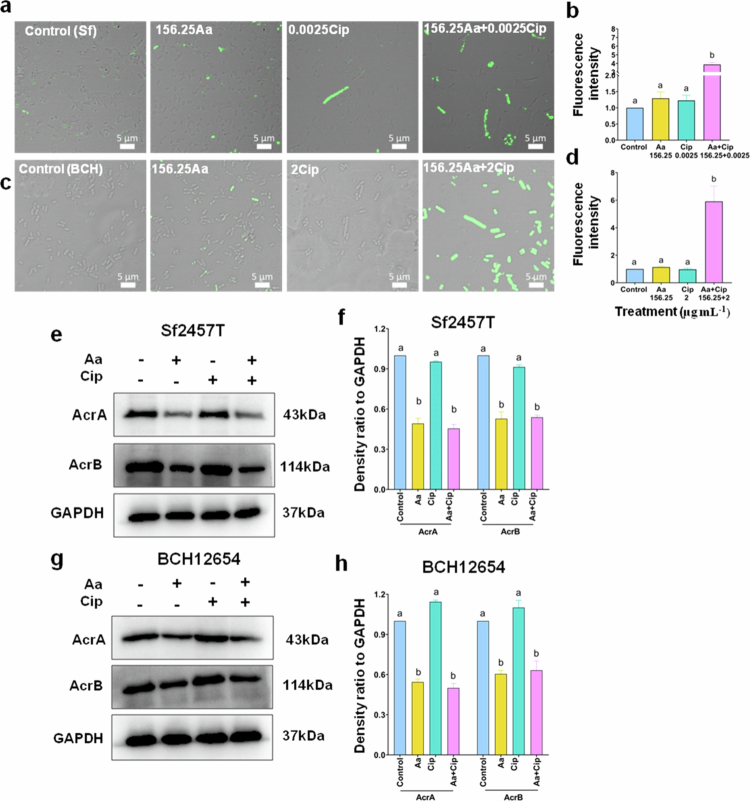
Inhibitory effects of asiatic acid (Aa) and ciprofloxacin (Cip) co-treatment on the multidrug efflux system of *S. flexneri. S. flexneri* (Sf2457T/BCH12654) cultures were treated with mono- or co-therapy of asiatic acid and ciprofloxacin. Confocal microscopy was used to visualize and quantify the membrane potential of treated *S. flexneri* using the fluorescent dye DiBAC4(3) (a–d). The expression of *S. flexneri* efflux proteins upon treatment were assessed by western blotting, and the density ratio normalized to that of GAPDH was graphically represented (e–h). All images are representative of three independent biological replicates, and the data are represented as mean ±S.E.M. Statistical significance was determined using ANOVA test (one-way). Results are statistically significant at *p* < 0.05. Group differences are marked by different superscript letters.

### Co-administration of asiatic acid and ciprofloxacin hampers *S. flexneri* membrane and nuclear integrity

Asiatic acid is reported to disrupt the bacterial membrane,^20^ whereas ciprofloxacin acts by blocking DNA gyrase activity, thereby preventing DNA replication and arresting cell division.^61^ Therefore, to understand surface and morphological changes, confocal and surface electron microscopy (SEM) imaging was performed. After treatment with subinhibitory concentration of ciprofloxacin, extensive filamentation was visualized for the standard strain, whereas only a few elongated cells were visualized in the clinical strain (Figures 6a–c, 7a–c). We quantified the filamentous phenotype (Supplementary Figure S6). Results showed that compared to untreated cells, an approximately 10-fold and 6-fold increase in the cell length of the standard strain was observed upon ciprofloxacin treatment in the absence or presence of Asiatic acid, respectively (Supplementary Figure S6a). However, the MDR strain did not show a significant alteration in cell length upon ciprofloxacin or co-treatment (Supplementary Figure S6b).

**Figure 9.**
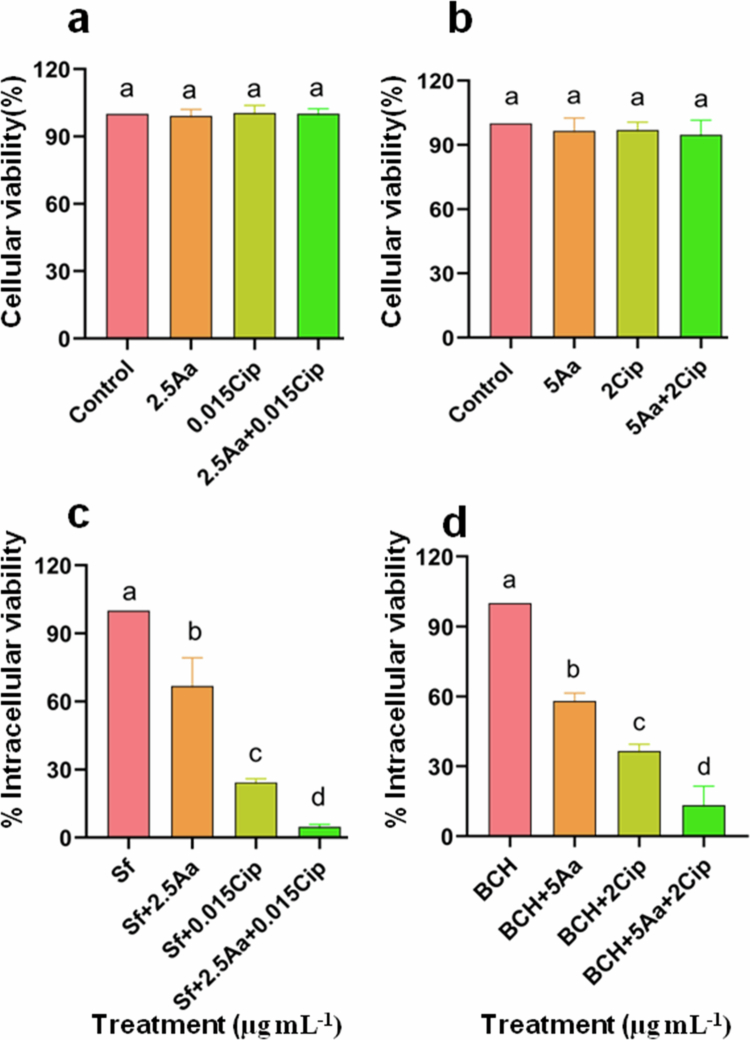
Effect of asiatic acid (Aa) and ciprofloxacin (Cip) co-treatment on *in vitro* safety and efficacy against *S. flexneri*. HT-29 cells (1 × 10^4^) were treated with mono or combination of asiatic acid with ciprofloxacin for 24 h, and cellular viability was assessed using MTT reagent and graphically represented as the % cell viability (a, b). HT-29 cells infected with *S. flexneri* (MOI 100) were treated with asiatic acid in the presence or absence of ciprofloxacin for 24 h, and the intracellular bacterial load was quantified by plate count method and graphically represented as % intracellular viability for Sf2457T (c) and the resistant BCH12654 strain (d). The data are presented as the mean of three independent biological replicates ±S.E.M. Statistical significance was determined using ANOVA test (one-way). Results are statistically significant at *p *< 0.05. Differences among groups are marked by different superscript letters.

Further live/dead imaging using SYTO9 and PI revealed that *S. flexneri* standard cells receiving monotherapy (only asiatic acid or ciprofloxacin) were live with intact membrane. Ciprofloxacin treatment, as reported caused cell division inhibition, resulting in the formation of filamentous rods, with the majority of the bacteria remaining viable (Figure 6). However, Asiatic plus ciprofloxacin treatment revealed that most of the filamentous cells elicited a marked increase in PI (red) staining thereby indicating increased inner membrane damage or cell death (Figure 6a,d). Furthermore, inner-membrane permeability was again assessed by spectrofluorimetric analysis of PI intake. Consistent with the live/dead imaging data, a marked increase in the intensity of PI fluorescence confirmed enhanced inner membrane permeability or a heavily compromised inner membrane upon combination therapy (Supplementary Figure S7a). Similarly, confocal imaging revealed similar results for the *S. flexneri* clinical isolate BCH12654 (Figure 7a,d). PI staining by spectrofluorimetric method also confirmed an increase in inner membrane permeability upon co-treatment (Supplementary Figure S7b). Next, we also determined membrane permeability by FM4-64/DAPI staining. The results revealed damaged or disrupted membranes upon asiatic acid plus ciprofloxacin co-treatment, as observed by the absence of FM4-64 staining (Figure 6b,e). Untreated or monotherapy-treated cells, however, showed FM4-64 staining, representing an intact membrane (Figure 6b,e). In coexistence, results were similar with clinical isolate BCH12654 (Figure 7b,e).

**Figure 10.**
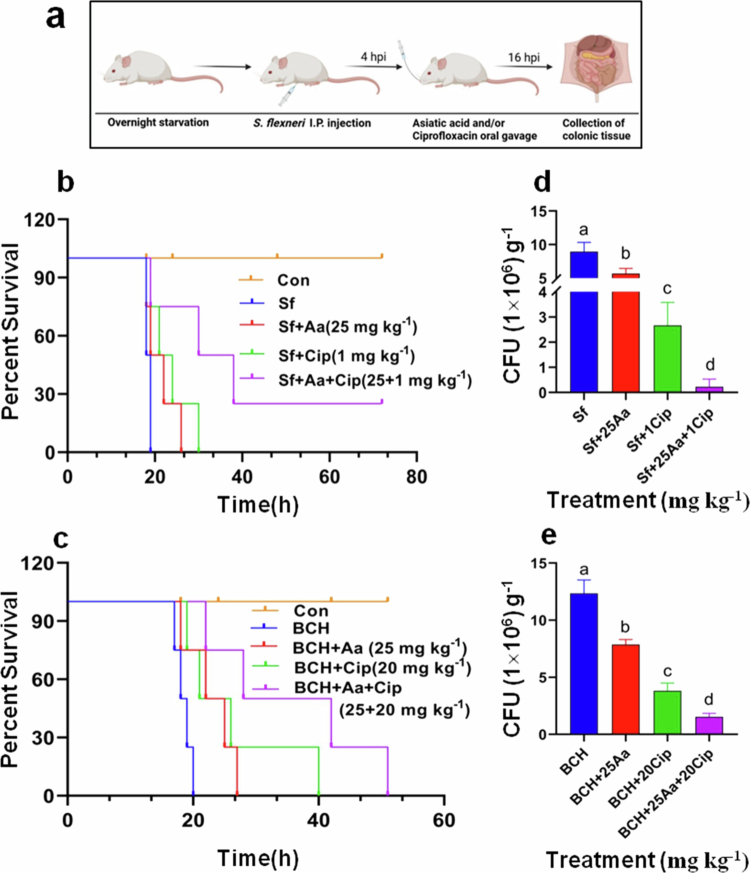
*In vivo* antimicrobial efficacy of asiatic acid (Aa) and ciprofloxacin (Cip) co-treatment against *S. flexneri* infection. Schematic representation of S. flexneri infection in mice model (a). Briefly, fasted BALB/c mice were intraperitoneally infected with *S. flexneri* (10^8^ CFU), followed by treatment with ciprofloxacin in the presence or absence of asiatic acid. Percent survival was monitored over the next 72 h, plotted and compared using Kaplan–Meier survival curve (b and c). Gentamicin treated mice colonic tissues were plated to determine bacterial colonization and are graphically represented as CFU g^−1^ of tissue (d and e). The data are presented as the mean of three independent biological replicates ±S.E.M. Statistical significance was determined using ANOVA test (one-way). Results are statistically significant at *p *< 0.05. Group differences are marked by distinct superscript letters.

To check whether co-treatment of *S. flexneri* (Sf2457T) with the compounds at higher synergistic dose [FICI 0.3125; (312.5Aa + 0.00125Cip)] yielded similar results, membrane assays (live/dead and FM4-64/DAPI) were carried out. Irrespective of the combinatorial dose (156.25Aa + 0.0025Cip or 312.5Aa + 0.00125Cip), the mechanism remains the same and comparable with observable increase in cell membrane damage (Supplementary Figure S8a,b,d,e).

Enhanced bacterial cell membrane permeability or disintegrity is associated with increased nucleic acid release. Simultaneously, we determined the leakage of nucleic acid by measuring the O.D. of the culture supernatant. Our data revealed significant leakage of nuclear material in the culture supernatant of co-treated cells of both standard and clinical strains (Supplementary Figure S7c,d). This leakage of nuclear material was also evident upon *in situ* DNA detection with TOTO-1 dye and agarose gel electrophoresis (Supplementary Figures S9, S10). Significant leakage of nucleic acid and the presence of eDNA were observed in co-treatment groups of both strains (Supplementary Figures S9, S10).

To further confirm bacterial membrane disruption, surface ultrastructure was studied using SEM imaging. Consistent with the above results, both standard and clinical strain control cells showed regular and intact surface morphology. As expected, asiatic acid and ciprofloxacin co-treated cells displayed signs of extensive membrane damage with ruptured cells. The presence of lysed cellular debris was also evident (Figures 6c, 7c). Quantification of the total number of ruptured or damaged cells was performed, showing increase in cell disintegration in the presence of co-treatment (Figures 6f, 7f). All together, these results indicate that the combination of asiatic acid and ciprofloxacin leads to bactericidal activity, which is associated with membrane rupture.

### Synergism modulates *S. flexneri* efflux system

Substantial evidences reported that bacterial membrane damage is highly associated with the proton motive force (PMF) required for ATP generation.^40^^,^^62^ Therefore, we checked the membrane potential (component of PMF) by using the fluorescent dye DiBAC4(3). Influx of the dye is increased in cells with hampered membrane potential or depolarized cells. Loss of membrane polarization was observed in bacteria treated with the synergistic combination of asiatic acid and ciprofloxacin as compared to control and monotherapy groups in both standard and clinical strain (Figure 8a‒d). Similar observation was noticed on co-treating *S. flexneri* (Sf2457T) with other synergistic doses (312.5Aa + 0.00125Cip) (Supplementary Figure S8c,f).

**Figure 11.**
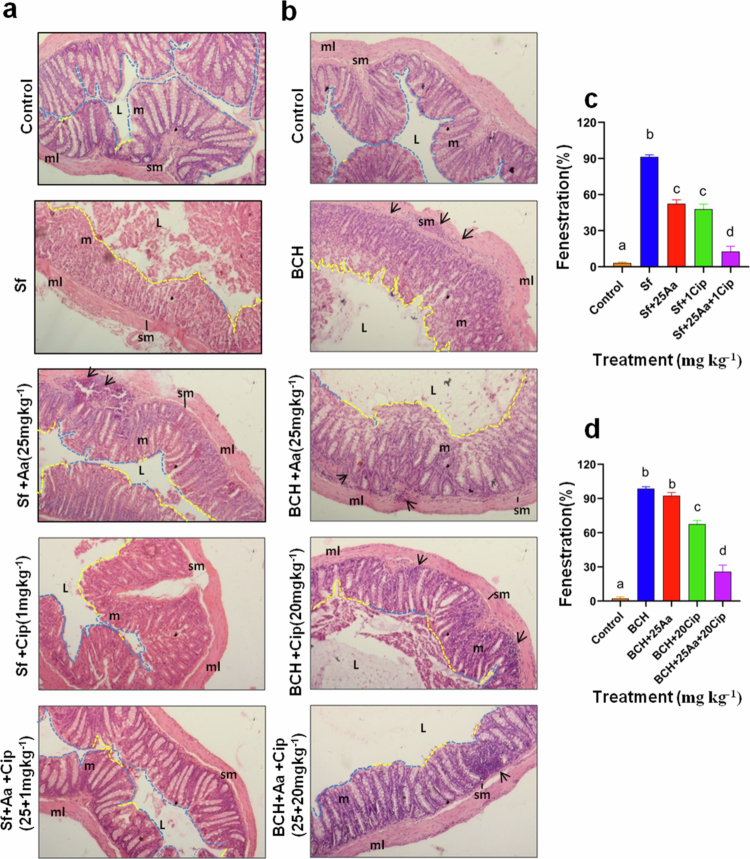
Colonic damage induced by *Shigella flexneri* is prevented by asiatic acid (Aa) and ciprofloxacin (Cip) co-treatment. Histopathological images of H&E stained colonic sections of mice under mono and co-treated conditions. Arrowhead indicates infiltration; L, lumen; sm, submucosa; m, mucosa; and ml, muscularis. Images were captured at 20× magnification (a and b). Percent epithelial fenestration across treatment groups was plotted. Blue and yellow dashed line indicates region with intact epithelium and regions with fenestration across colonic section respectively (c and d). Data are presented as mean of three independent biological replicates ±S.E.M. Statistical significance was determined using ANOVA test (one-way). Results are statistically significant at *p* < 0.05. Statistical differences across groups are marked by different superscript letters.

PMF is essential for multidrug transporters like efflux pump. So, inhibition of antibiotic efflux may be another probable cause of the synergy between asiatic acid and ciprofloxacin. The PMF-driven AcrAB‒TolC multidrug efflux system aids in antibiotic resistance.^63^ Moreover, the efflux pumps play major role in bacterial pathogenesis and virulence, including biofilm formation.^64^ Henceforth, we first checked the role of asiatic acid as an efflux pump inhibitor (EPI) through molecular docking studies. The interaction of asiatic acid with ciprofloxacin-bound inner membrane AcrB and the periplasmic AcrA protein of the efflux system were checked and compared to efflux inhibitor phenylalanine arginine β-naphthylamide (PAβN). The hinge site and membrane proximal (MP) site of AcrA play important role in the functionality of the pump. Here, we observed that both asiatic acid and PAβN are docked in a similar location at both the sites (Hinge and MP site) of ciprofloxacin-bound AcrA (Supplementary Figure S11a–d). Both asiatic acid and PAβN interacted with GLN 71 residue at the hinge site (Supplementary Figure S11a,b) and GLN 341 at the MP site (Supplementary Figure S11c,d). However, compared to the hinge site, the binding of asiatic acid at the MP site was more stabilized with three hydrogen and three hydrophobic interactions (Supplementary Figure S11a,c). Moreover, the binding energy of asiatic acid (−7) at MP site was comparable to that of PAβN (−7.2) (Supplementary Table S3). Similarly, both compounds share common interactions with Thr 37 and Leu 293 residues of AcrB (Supplementary Figure S11e,f) and form extensive hydrogen and hydrophobic bonds with binding energies of −8.3 and −9 for Aa and PAβN, respectively (Supplementary Table S3).

Next, the expression of acrA and acrB genes involved in antibiotic resistance of *S. flexneri* was checked at transcriptional level (Supplementary Figure S12). Over-expression of both efflux genes was observed in the resistant strain (BCH12654) compared to standard strain (Supplementary Figure S12a and b).

Further, immunoblot analysis was performed to compare the expression of efflux pump (AcrA/B) proteins under treatment conditions. Compared to control and ciprofloxacin treatments, significant downregulation of AcrA and AcrB was observed upon asiatic acid treatment and co-treatment in the presence of ciprofloxacin for both the bacterial strains (Figure 8e–h).

Finally, the inhibition of bacterial efflux activity was monitored in the presence of asiatic acid. The fluorescent dye EtBr is a common substrate of many efflux systems and is used for examining the activity of these pumps. No significant change in the efflux activity of the susceptible strain was observed upon asiatic acid addition (Supplementary Figure S13a). In contrast, the resistant strain overexpressing the acrA and acrB transcript showed decrease in efflux activity with increasing asiatic acid concentration compared to untreated cells. Known efflux inhibitor PAβN showed the lowest efflux activity (Supplementary Figure S13b). Collectively, the data suggest that asiatic acid in combination with ciprofloxacin are attributed to the loss of bacterial PMF and simultaneously efflux pump activity.

### Combination of asiatic acid with ciprofloxacin exhibited selective synergistic toxicity against bacteria

The result of asiatic acid and ciprofloxacin combination on intracellular bacterial survivability and cellular viability were investigated. The entero-invasive pathogen *S. flexneri* colonizes the gut of mammals, including humans. Therefore, the colonic epithelial cell line HT29 was used for *in vitro* studies.

The results showed that no significant loss of cellular viability was observed upon co-administration of asiatic acid and ciprofloxacin at different doses compared to drug monotherapy with asiatic acid and ciprofloxacin alone (Figure 9a, b). Further, invasion assay revealed a significant reduction in the intracellular burden of both bacterial strains upon co-treatment with the above doses of the compounds in combination compared to either drug alone (Figure 9c, d). So, Aa–Cip combination has high selective toxicity on bacteria over mammalian cells.

In addition, we checked other antibiotic and asiatic acid combination pairs. Cellular viability and infection assay were performed with other combinations (asiatic acid–ampicillin, asiatic acid–chloramphenicol and asiatic acid–nalidixic acid), which showed synergistic interaction based on FICI against both the *S. flexneri* strains (Supplementary Table S2). Cellular viability data revealed that the selected doses of these antibiotics and asiatic acid alone or in combination did not cause any significant observable changes in HT29 cell viability (Supplementary Figures S14a–c, S15a–c).

As for antimicrobial efficacy, the asiatic acid‒ampicillin pair significantly reduced the proliferation of only the standard strain but not the resistant strain when compared to the antimicrobial agents alone (Supplementary Figures S14d, S15d). Asiatic acid–chloramphenicol combination inhibited the intracellular load of both strains, but the reductions were insignificant as compared to chloramphenicol treatment alone (Supplementary Figures S14e, S15e). Interestingly, a significant reduction in the intracellular proliferation of both the bacterial strains in HT29 cells was observed upon co-treatment of asiatic acid with another quinolone antibiotic, nalidixic acid (Supplementary Figures S14f, S15f). Thus, the overall results suggest that asiatic acid may potentiate the activity of quinolone antibiotics (ciprofloxacin and nalidixic acid) *in vitro* and that the combination of asiatic acid with quinolone antibiotics showed selective toxicity toward bacteria over intestinal cells.

### Asiatic acid potentiates quinolone antibiotics *in vivo*

The anti-*shigella* effect of asiatic acid–antibiotic combination was also observed in mice model of shigellosis (Figure 10). A schematic presentation of the *in vivo* study is illustrated in Figure 10a, where the mice were subjected to intraperitoneal infection, followed by mono- or co-therapy with asiatic acid and antibiotic. Immunofluorescence imaging revealed the presence of *S. flexneri* in the epithelial layers of the colon (Supplementary Figure S16). We next checked the survival kinetics of the mice. Co-administration of asiatic acid with ciprofloxacin prevented the death of mice as compared with mono treatment groups. 100% death of the mice was noted within 19 h of infection with *S. flexneri* Sf2457T in the group that received no treatment. Infected (Sf2457T) mice treated with a combination of asiatic acid and ciprofloxacin showed a survival advantage of 50% at 30 h and 25% at 72 h, compared to group receiving either asiatic acid or ciprofloxacin (Figure 10b). As for clinical strain, 50% and 25% survival advantage of combination group was observed compared to groups receiving either asiatic acid or ciprofloxacin, respectively during 28 h. Additionally, 25% survivability was monitored during 42 h of co-treatment (Figure 10c).

Herein, we evaluated the combined efficacy of asiatic acid–ciprofloxacin combination. *Shigella* infection is primarily localized in gastrointestinal tissues, particularly the large intestine/colon. Therefore, bacterial colonization in the large intestine was checked. Compared to either antimicrobial agent alone, a significant reduction in the colonic bacterial burden was observed upon treatment with 25 + 1 mg kg^−1^ or 25 + 20 mg kg^−1^ of asiatic acid plus ciprofloxacin for standard and BCH12654 strains, respectively (Figure 10d, e).

Although, rare (0.4%−7.3%), but *Shigella* can enter the bloodstream in humans, with majority of cases caused by *S. flexneri.*^65^^,^^66^ Such reports are also available in an intraperitoneal mice model of infection.^67^ Therefore, we next checked for the presence of *S. flexneri* (Sf2457T) in the systemic organs (liver and spleen). In accordance with a previous report, we observed *S. flexneri* in the bloodstream of mice as early as 4 h after intraperitoneal infection, and the load increased over time (Supplementary Figure S17a). Additionally, the bacterial load was detected in the liver and spleen (Supplementary Figure S17b,c). Compared to either antimicrobial agents, co-therapy resulted in a significant reduction in bacterial colonization in the liver (Supplementary Figure S17b). In the splenic tissue, significant lowering of bacterial load in the co-treatment group was observed compared to untreated and asiatic acid alone. Although the bacterial load was lowered in the co-treatment group, compared to ciprofloxacin treatment it was not significantly lowered (Supplementary Figure S17c). As intraperitoneal administration of *S. flexneri* resulted in systemic infection, we further confirmed the bacterial population in the blood via intravenous infection (Supplementary Figure S18). Similarly, oral administration of Aa‒Cip combination treatment showed reduced bacterial count in the blood (Supplementary Figure S18).

Both ciprofloxacin and nalidixic acid share similar antibacterial mechanism of action. We additionally, checked if asiatic acid–nalidixic acid combination could mimic similar protective efficacy *in vivo*. In line, asiatic acid–nalidixic acid combination significantly decreased bacterial intestinal colonization compared to monotherapy and infection control (Supplementary Figure S19). Collectively, all these results demonstrate that asiatic acid effectively restores the efficacy of the quinolone group of antibiotics ciprofloxacin and nalidixic acid against standard and clinical isolate of *S. flexneri*.

Next, the *in vivo* activity of the Asiatic acid‒ciprofloxacin combination was assessed by histopathological examination of the colon. The normal architecture of the colon, as observed in the control groups, was completely destroyed in the infected mice groups of both *S. flexneri* strains (Sf2457T and BCH12654) (Figure 11a,b). Mice infected with both strains showed extensive colonic epithelial erosion and fenestration (Figure 11a–d**)**. The infiltration of the mucosa and submucosa was more evident in the resistant strain (Figure 11b). However, infected mice treated with monotherapy of asiatic acid or ciprofloxacin, showed minor recovery from tissue damage. Combined treatment with asiatic acid and ciprofloxacin resulted in better protection of the colonic tissue against infection, with an intact mucosal lining and reduced fenestration (Figure 11a–d). Additionally, antimicrobial agent co-administration in infection-free mice showed intact epithelium without intestinal tissue damage (Supplementary Figure S20a,b). These data revealed that the synergistic dose of asiatic acid and ciprofloxacin combination is nontoxic to uninfected mice. Collectively, all these results demonstrate that asiatic acid effectively restores the efficacy of the quinolone group of antibiotics ciprofloxacin and nalidixic acid against standard and clinical isolates of *S. flexneri*.

## Discussion

The increasing incidence of death due to drug-resistant infections, majorly originating from Gram-negative bacteria, threatens the world as an epidemic.^68^ Multiple factors drive the development of resistance and the emergence of multidrug-resistant infections. *S. flexneri*, the pathogen responsible for bacillary dysentery, causes significant morbidity and mortality majorly among children and immunocompromised individuals.^1^^,^^2^ Although few antibiotics are in the pipeline to treat this bacterial pathogen, the WHO has recommended the urgent requirement for developing novel and alternative strategies to combat the emergence of MDR *S. flexneri* infections.^9^ In comparison to the search for new antibiotics, an adjuvant strategy is considered as an effective and economical approach to tackle MDR infections. Hence, combinatorial drug pairs are more efficient to fight AMR but are scarcely used in treatments and remain mostly unexplored. Combining different small molecules or herbal compounds with antibiotics is a promising strategy for increasing treatment efficacy, reducing toxicity and evolution of resistance.^15^^,^^69^

Several compounds from natural sources, such as Capsaicin, Caffeine, Thymol, Carvacrol, Quercetin, and others are reported to act as antibiotic potentiators.^15^^,^^69^ Asiatic acid, a pentacyclic triterpenoid, is not well explored in terms of infection therapeutics despite the presence of a large database of this group of molecules as an anti-infective agent.^18–22,70^ Earlier, we have reported that asiatic acid induces antimicrobial peptide production in intestinal cells to inhibit intracellular *S. flexneri* growth.^23^ Herein, for the first time, we report that asiatic acid acts as an antibiotic adjuvant to inhibit *S. flexneri* growth both *in vitro* and *in vivo*. Asiatic acid restored ciprofloxacin activity against both standard and clinical MDR isolates of *S. flexneri*. It also potentiates another quinolone antibiotic nalidixic acid to act against *S. flexneri*. In particular, the adjunct activity of asiatic acid to ciprofloxacin was robust, with a significantly lower FICI. Similar to our findings, a previous study reported that combination of Clerodane diterpene, a bicyclin diterpene isolated from leaves of *Polyalthia longifolia* together with fluoroquinolone norfloxacin, ciprofloxacin and ofloxacin synergistically inhibited the growth of Methicillin resistant *Staphylococcus aureus* MRSA.^71^ Moreover, we have also observed similar synergistic interaction between asiatic acid with other bactericidal antibiotic ampicillin but not streptomycin. Azithromycin and tetracycline, however, showed additive or antagonist interactions with asiatic acid. However, an exception was observed in combining bactericidal compound asiatic acid with the bacteriostatic antibiotic chloramphenicol against *S. flexneri*. Although the combination of bacteriostatic and bactericidal antimicrobials mostly results in antagonistic interaction, exceptions have been reported and are used for treating bacterial infections.^72^ Moreover, the variations observed in the MIC of asiatic acid against the *S. flexneri* strains was insignificant, probably indicating disruption of some vital cellular components as a probable mechanism of action.^20^ The fact that asiatic acid and ciprofloxacin co-therapy were effective against several clinical isolates further indicated that they acted on additional mechanisms different from the existing mode of action of antibiotics or asiatic acid alone. Most importantly, the addition of asiatic acid prevented the evolution of bacterial resistance to ciprofloxacin.

The cell membrane is a vital component providing protective barrier and controlling essential functions, including the transport and energy production required for bacterial survival.^73^ In this regard, our mechanistic studies demonstrated that the combination of asiatic acid with ciprofloxacin disrupted the bacterial membrane, accompanied by perturbation of the membrane potential essential for carrying out cellular functions. These mechanisms explain the synergistic action of asiatic acid with ciprofloxacin against *S. flexneri*. In line with our observations, a previous study showed that asiatic acid caused membrane disruption of the gut bacteria *Clostridium difficile.*^20^

Another important factor that drives drug synergy is the modulation of bacterial efflux pumps. Although these pumps are expressed in antibiotic-susceptible strains, their overexpression is associated with resistance phenotype.^74^ Among the many factors that inhibit efflux pumps,^75^we found significant downregulation of AcrAB-TolC pump proteins (AcrA and AcrB) upon co-treatment in both the bacterial strains. However, the efflux activity of only the resistant strain was inhibited upon asiatic acid treatment. Therefore, an active efflux pump contributes to the loss of susceptibility of the strain (BCH12654) to ciprofloxacin. Synergism also reduced PMF, which in turn probably impaired the function of efflux pumps that use PMF to export antibiotics, thus restoring the susceptibility of the resistant strain to ciprofloxacin. PMF is also essential for ATP synthesis and nutrient transport.^62^ Thus, lowering of PMF could probably also decrease the viability of both the strains. Despite our knowledge about drug–antibiotic interactions, fundamental questions about mechanistic details remain unanswered. Furthermore, biofilm plays a pivotal role in establishing infection and its recurrence. During its passage through the small intestine, *S. flexneri* encounters bile. These bile salts induce biofilm formation and trigger the expression of proteins related to virulence, adhesion and the efflux system. Altogether these factors facilitate successful bacterial passage to the colon, better disease manifestation and development into resistant strain.^33^^,^^58^ Herein, we observed that ciprofloxacin in combination with asiatic acid hinders biofilm formation better than antibiotic alone. Previous reports also indicated that asiatic acid and other terpenoids disrupt biofilms and increase the susceptibility of biofilms to antibiotics, which supports our findings.^24^^,^^25^ Interestingly, the response of *S. flexneri* planktonic and biofilm cells to mono- or co-therapy with asiatic acid is different. Planktonic cells exposed to asiatic acid (mono- or co-therapy) showed membrane disruption, causing significant leakage of nuclear material (DNA) and resulting in cell death. On the other hand, under similar treatment conditions, the eDNA content of the biofilm matrix is decreased. *S. flexneri* biofilm matrix is formed of exopolysaccharides, eDNA, proteins and lipids, which form a structured biofilm community.^76^ Decrease in exopolysaccharide and eDNA contents were observed upon exposure to asiatic acid in the presence or absence of ciprofloxacin, which arrested biofilm formation. Similar to our study, pentacyclic triterpenoid compounds (glycyrrhetinic acid, ursolic acid and betulinic acid) hindered *Vibrio cholera* biofilm formation by inhibiting matrix (carbohydrate, eDNA, protein, and lipid) production.^77^

Thus, overall co-treatment not only inhibited *S. flexneri* planktonic growth but could also prevent secondary infections arising from biofilm. Similar planktonic growth and biofilm inhibitory mechanisms were shown by *S. flexneri* in response to ferulic acid treatment.^76^

Another important observation in our study is the selective toxicity towards bacteria rather than mammalian cells. Asiatic acid–ciprofloxacin combination killed intracellular *S. flexneri* but was nontoxic towards intestinal cells. Similar result was mimicked by asiatic acid–nalidixic acid combination. Asiatic acid is reported to have immunomodulatory effect on mammalian cells and also shows anti-cancer activity.^78^ Therefore, this selective toxicity toward bacteria but not mammalian cells is a crucial factor for the application of this synergistic composition against intracellular bacteria. Additionally, bacterial invasion/intracellular growth and biofilm formation are associated with virulence factors.^33^^,^^58^ Therefore, asiatic acid in combination with ciprofloxacin probably reduces bacterial virulence factors and thereby inhibits bacterial proliferation along with biofilm formation. Probably, these multifactorial mechanisms resulted in the loss of bacterial viability.

Finally, we validated the synergistic role of the asiatic acid antibiotic combination in an intraperitoneal mice model of *S. flexneri*. The combination effect lowered the bacterial count in systemic (liver, spleen) and mucosal (large intestine) tissue and repaired intestinal tissue damage. The synergistic composition of both asiatic acid–ciprofloxacin and asiatic acid–nalidixic acid was able to combat *S. flexneri* infection, proving the ability of asiatic acid to increase the efficacy of the quinolone group of antibiotics. Co-therapy also inhibited the bacterial count in the blood during intravenous infection. In addition, asiatic acid–ciprofloxacin co-treatment has no effect on uninfected mice, further proving selective toxicity for bacteria.

However, considerable clinical data are necessary to examine the clinical outcome of asiatic acid–ciprofloxacin combination and its probable complications in humans. In summary, we showed that asiatic acid remarkably restored the functional ability of ciprofloxacin by inducing membrane damage while inhibiting biofilm formation and efflux activity. Importantly, asiatic acid slows down the resistance development, and when combined with ciprofloxacin, it effectively reduces *S. flexneri* infection *in vivo*, emphasizing its therapeutic ability as a potential nonantibiotic adjuvant.

## Supplementary Material

Supplementary material Supplementary figures and tables.

## Data Availability

All figures and tables supporting the findings of the study are available within the article and Supplementary Material.
