# Supplementary material for: Asiatic acid, a novel ciprofloxacin adjuvant inhibits Shigella flexneri infection
Source: Gut Microbes. 2025 Nov 30;17(1):2586329. doi: 10.1080/19490976.2025.2586329 (PMC12667651; doi:10.1080/19490976.2025.2586329)
Supplement: Supplementary material — Supplementary figures and tables. [file KGMI_A_2586329_SM1596.docx]

**Supplementary Table S1. Antibiotic resistance profile of multidrug resistant clinical isolates of *Shigella flexneri***

| Bacteria | ID of isolates | Antibiotic resistance pattern |
| --- | --- | --- |
| *Shigella flexneri* serotype 2a | BCH12654 | NA, CIP, NOR, OFX, TET, S, AM, E, CHL |
|  | IDH10994 | NA, CIP, NOR, OFX, S, AM, E, SXT, CHL |
|  | IDH14377 | NA, CIP, NOR, OFX, TET, S, AM, E, SXT |
|  | IDH14351 | NA, CIP, NOR, OFX, TET, S, AM, E, SXT |
|  | IDH14386 | NA, CIP, NOR, OFX, TET, S, AM, E, SXT |
|  | IDH14363 | NA, CIP, NOR, OFX, S, AM, E, SXT, CHL |
|  | IDH10932 | NA, CIP, NOR, OFX, TET, S, AM, E, SXT |

**Supplementary Table S2. *In-vitro* interaction between Asiatic acid (Aa) and Antibiotics against *Shigella flexneri* strains depicted by Fractional Inhibitory Concentration Indices (FICI)**

| *S. flexneri* | Compound | | MIC alone (µgmL^-1^)  MIC A MIC B | | MIC in combination (µgmL^-1^)  MIC A MIC B | |  | |
| --- | --- | --- | --- | --- | --- | --- | --- | --- |
|  | A B | |  |  |  |  |  |  |
|  |  |  |  |  |  |  | FICI Interpretation | |
| **Sf2457T** | Asiatic acid | Ampicillin | 1250 | 7.8 | 156.25 | 1.95 | 0.374 | Synergy |
|  | Asiatic acid | Chloramphenicol | 1250 | 8 | 312.5 | 2 | 0.5 | Synergy |
|  | Asiatic acid | Ciprofloxacin | 1250 | 0.02 | 156.25 | 0.0025 | 0.25 | Synergy |
|  | Asiatic acid | Nalidixic acid | 1250 | 15.6 | 156.25 | 3.9 | 0.375 | Synergy |
|  | Asiatic acid | Streptomycin | 1250 | 7.8 | 78.125 | 7.8 | 1.062 | Additive |
|  | Asiatic acid | Tetracycline | 1250 | 0.24 | 625 | 0.24 | 1.5 | Additive |
|  | Asiatic acid | Azithromycin | 1250 | 0.97 | 1250 | 0.97 | 2.0 | Antagonist |
| **BCH12654** | Asiatic acid | Ampicillin | 1250 | 1000 | 156.25 | 125 | 0.25 | Synergy |
|  | Asiatic acid | Chloramphenicol | 1250 | 1000 | 156.25 | 250 | 0.375 | Synergy |
|  | Asiatic acid | Ciprofloxacin | 1250 | 8 | 156.25 | 2 | 0.375 | Synergy |
|  | Asiatic acid | Nalidixic acid | 1250 | 500 | 312.5 | 125 | 0.5 | Synergy |
|  | Asiatic acid | Streptomycin | 1250 | 250 | 156.25 | 250 | 1.125 | Additive |
|  | Asiatic acid | Tetracycline | 1250 | 31.25 | 78.125 | 31.25 | 1.0625 | Additive |
|  | Asiatic acid | Azithromycin | 1250 | 1.95 | 78.125 | 0.975 | 0.562 | Additive |
| **IDH10994** | Asiatic acid | Ampicillin | 1250 | 1000 | 78.125 | 250 | 0.31 | Synergy |
|  | Asiatic acid | Chloramphenicol | 1250 | 250 | 156.25 | 62.5 | 0.375 | Synergy |
|  | Asiatic acid | Ciprofloxacin | 1250 | 11 | 78.125 | 2.75 | 0.31 | Synergy |
|  | Asiatic acid | Nalidixic acid | 1250 | 4000 | 39.06 | 2000 | 0.531 | Additive |
|  | Asiatic acid | Streptomycin | 1250 | 250 | 78.125 | 125 | 0.562 | Additive |
|  | Asiatic acid | Tetracycline | 1250 | 0.24 | 78.125 | 0.24 | 1.062 | Additive |
|  | Asiatic acid | Azithromycin | 1250 | 0.97 | 78.125 | 0.97 | 1.062 | Additive |
| **IDH14386** | Asiatic acid | Ampicillin | >1250 | 1000 | 312.5 | 500 | 0.625 | Additive |
|  | Asiatic acid | Chloramphenicol | >1250 | 4 | 78.125 | 2 | 0.531 | Additive |
|  | Asiatic acid | Ciprofloxacin | >1250 | 46 | 78.125 | 11.5 | 0.281 | Synergy |
|  | Asiatic acid | Nalidixic acid | >1250 | 4000 | 78.125 | 2000 | 0.531 | Additive |
|  | Asiatic acid | Streptomycin | >1250 | 500 | 2500 | 500 | 2.0 | Antagonist |
|  | Asiatic acid | Tetracycline | >1250 | 31.25 | 156.25 | 15.625 | 0.562 | Additive |
|  | Asiatic acid | Azithromycin | >1250 | 62.5 | 2500 | 62.5 | 2.0 | Antagonist |
| **IDH10932** | Asiatic acid | Ampicillin | 1250 | 1000 | 312.5 | 250 | 0.5 | Synergy |
|  | Asiatic acid | Chloramphenicol | 1250 | 4 | 78.125 | 2 | 0.56 | Additive |
|  | Asiatic acid | Ciprofloxacin | 1250 | 11 | 78.125 | 5.5 | 0.562 | Additive |
|  | Asiatic acid | Nalidixic acid | 1250 | 4000 | 39.06 | 2000 | 0.531 | Additive |
|  | Asiatic acid | Streptomycin | 1250 | 500 | 1250 | 500 | 2.0 | Antagonist |
|  | Asiatic acid | Tetracycline | 1250 | 31.25 | 78.125 | 31.25 | 1.062 | Additive |
|  | Asiatic acid | Azithromycin | 1250 | 62.5 | 1250 | 62.5 | 2.0 | Antagonist |
| **IDH14363** | Asiatic acid | Ampicillin | 1250 | 1000 | 39.06 | 250 | 0.281 | Synergy |
|  | Asiatic acid | Chloramphenicol | 1250 | 500 | 156.25 | 250 | 0.625 | Additive |
|  | Asiatic acid | Ciprofloxacin | 1250 | 11 | 78.125 | 2.75 | 0.312 | Synergy |
|  | Asiatic acid | Nalidixic acid | 1250 | 4000 | 156.25 | 1000 | 0.375 | Synergy |
|  | Asiatic acid | Streptomycin | 1250 | 500 | 78.125 | 250 | 0.562 | Additive |
|  | Asiatic acid | Tetracycline | 1250 | 0.97 | 78.125 | 0.485 | 0.562 | Additive |
|  | Asiatic acid | Azithromycin | 1250 | 0.97 | 1250 | 0.97 | 2.0 | Antagonist |
| **IDH14351** | Asiatic acid | Ampicillin | 1250 | 4000 | 39.06 | 4000 | 1.031 | Additive |
|  | Asiatic acid | Chloramphenicol | 1250 | 4 | 156.25 | 2 | 0.625 | Additive |
|  | Asiatic acid | Ciprofloxacin | 1250 | 11 | 312.5 | 2.75 | 0.5 | Synergy |
|  | Asiatic acid | Nalidixic acid | 1250 | 4000 | 156.25 | 500 | 0.25 | Synergy |
|  | Asiatic acid | Streptomycin | 1250 | 1000 | 1250 | 2000 | 3.0 | Antagonism |
|  | Asiatic acid | Tetracycline | 1250 | 15.6 | 78.125 | 15.6 | 1.062 | Additive |
|  | Asiatic acid | Azithromycin | 1250 | 3.9 | 2500 | 3.9 | 3.0 | Antagonism |
| **IDH14377** | Asiatic acid | Ampicillin | 1250 | 4000 | 312.5 | 2000 | 0.75 | Additive |
|  | Asiatic acid | Chloramphenicol | 1250 | 4 | 39.06 | 1 | 0.281 | Synergy |
|  | Asiatic acid | Ciprofloxacin | 1250 | 5.5 | 78.125 | 2.75 | 0.562 | Additive |
|  | Asiatic acid | Nalidixic acid | 1250 | 4000 | 156.25 | 1000 | 0.375 | Synergy |
|  | Asiatic acid | Streptomycin | 1250 | 1000 | 625 | 1000 | 1.5 | Additive |
|  | Asiatic acid | Tetracycline | 1250 | 15.6 | 78.125 | 15.6 | 1.062 | Additive |
|  | Asiatic acid | Azithromycin | 1250 | 3.9 | 1250 | 3.9 | 2.0 | Antagonist |


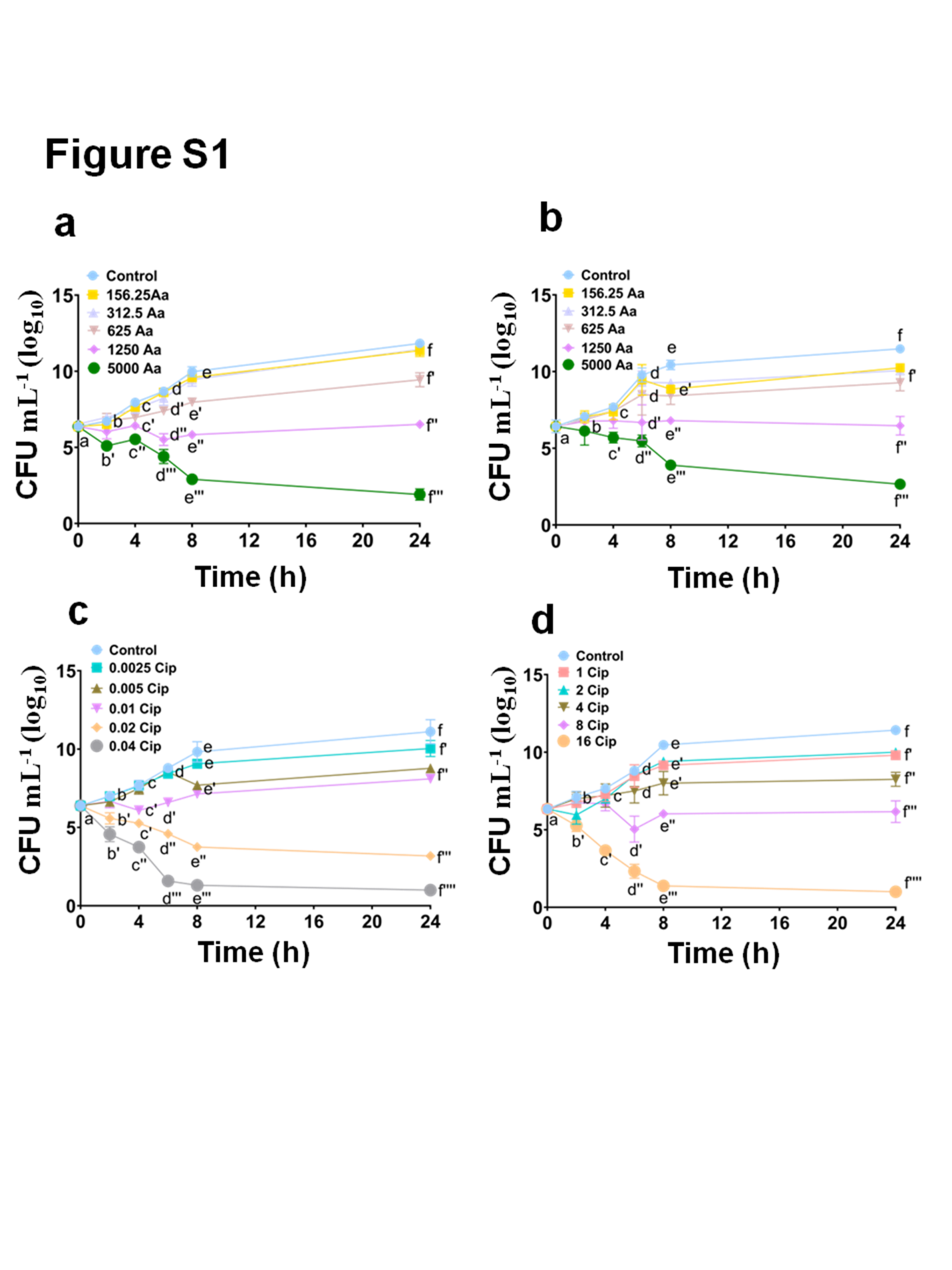


**Supplementary Figure S1. Growth of *Shigella flexneri* in presence of Asiatic acid and Ciprofloxacin monotherapy**. 5× 10^5^ CFU mL^-1^ of *S. flexneri* (Sf2457T/BCH12654) cultures were grown in presence of either Asiatic acid (5000-156.25 µgmL^-1^) or Ciprofloxacin (0.02-0.000625 or 8-0.25 µgmL^-1^). Time-dependent killing curves were assessed by CFU count and plotted as log_10_ CFU for Asiatic acid (**a-b**) and Ciprofloxacin (**c-d**). Data are representative of three independent biological replicates and represented as +S.E.M. Statistical significance was evaluated by performing Two-way ANOVA. Results are statistically significant at *p* <0.05. Statistical difference among groups are denoted by different superscript letters


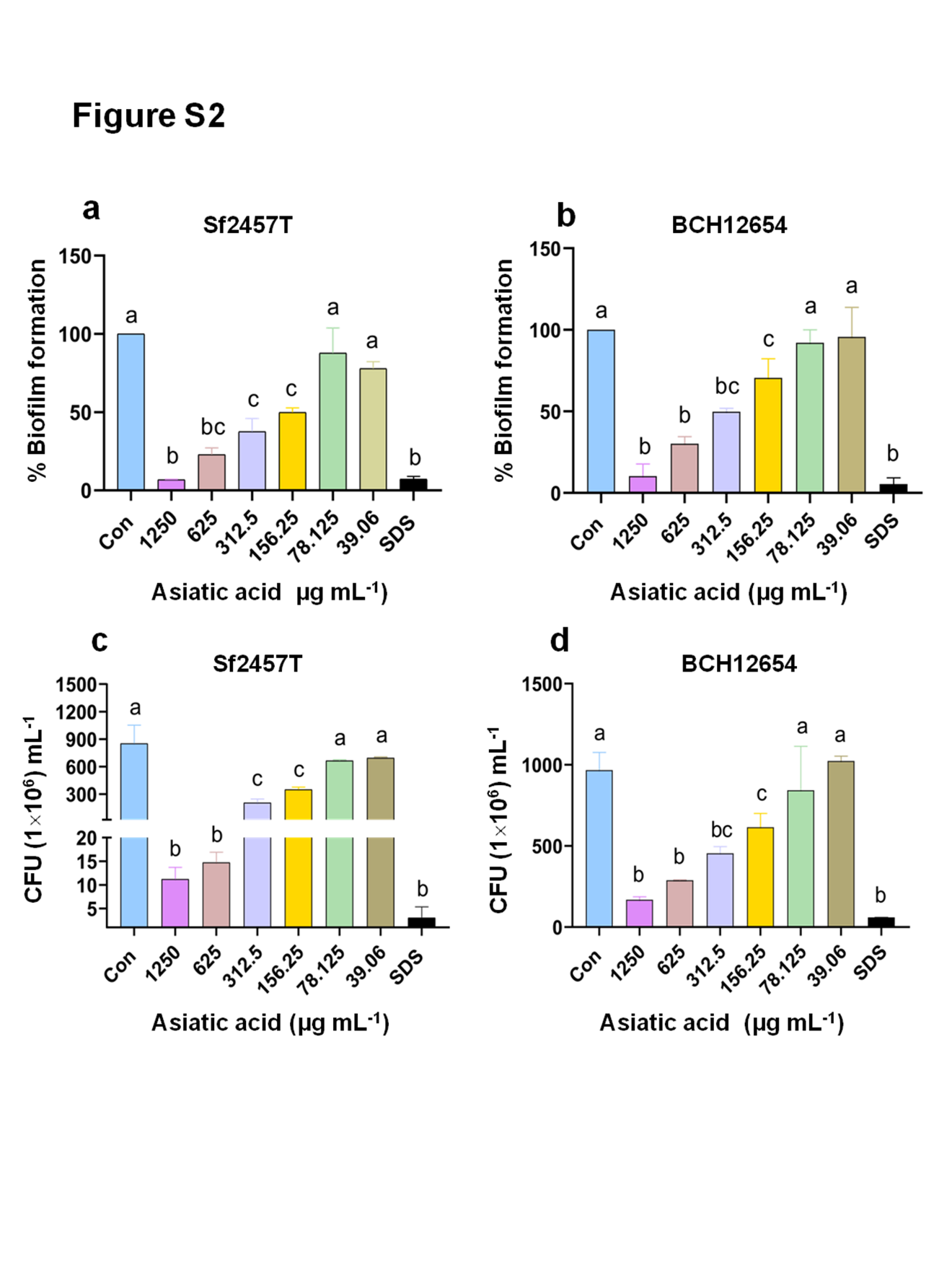


**Supplementary Figure S2. Asiatic acid (Aa) treatment inhibited biofilm formation by *S. flexneri***. *S. flexneri* (Sf2457T/BCH12654) cultures were grown in presence of Asiatic acid (1250-39.06 µgmL^-1^) for 24 h in biofilm inducing conditions (1.2% bile salt). The inhibition of biofilm formation was quantified by measuring the O.D at 540nm of the crystal violet stained biofilms and graphically represented as % Biofilm formation (**a-b**) and biofilm viabilitywas assessed by plate count method and represented as CFU mL^-1^ (**c-d**). Data are representative of three independent biological replicates and represented as +S.E.M. Statistical significance was evaluated by performing One-way ANOVA. Results are considered statistically significant at *p* <0.05. Differences among groups are marked by different superscript letters.for


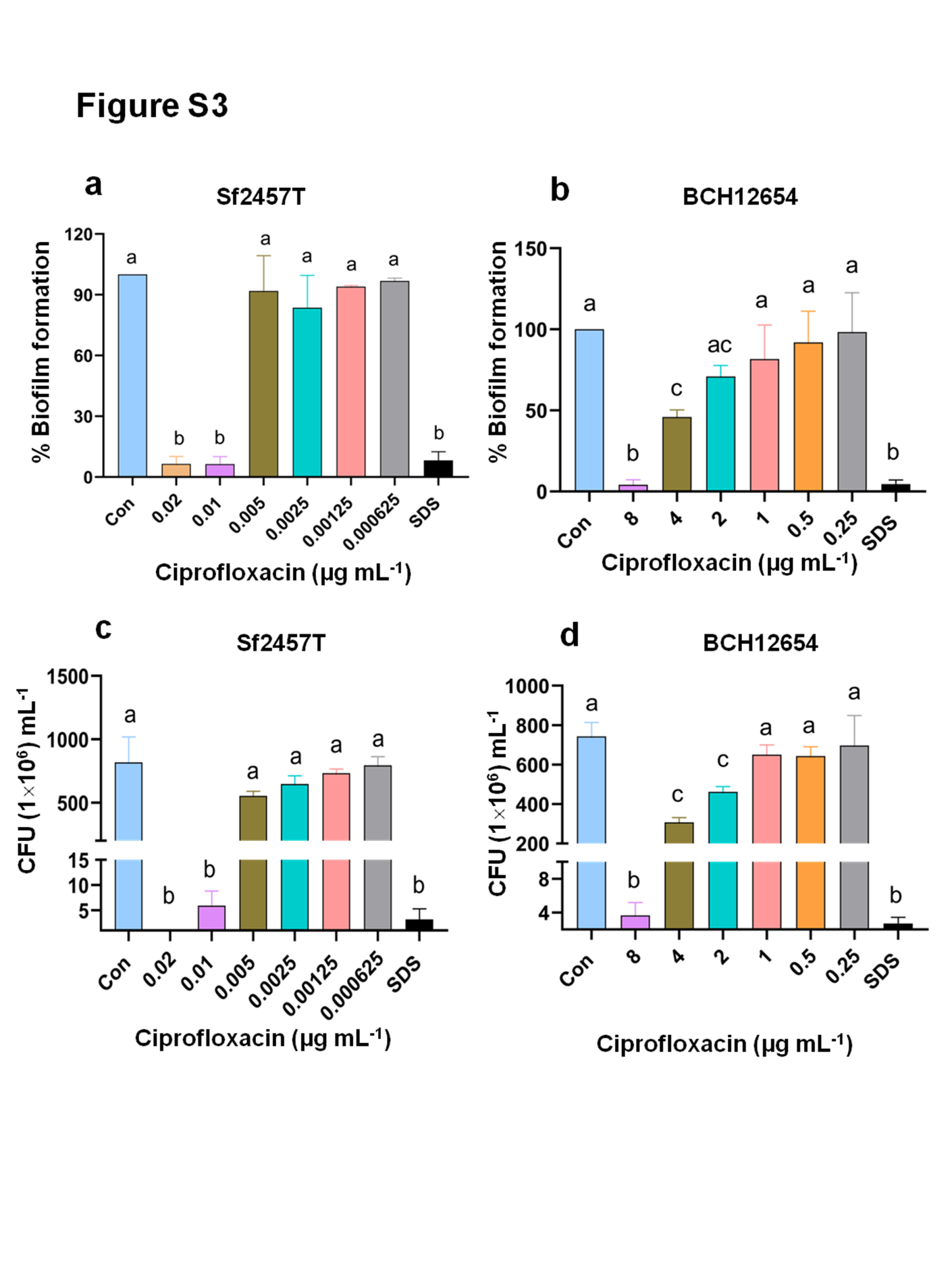


**Supplementary Figure S3. Changes in biofilm formation by *S. flexneri* with Ciprofloxacin treatment**. *S. flexneri* (Sf2457T/ BCH12654) cultures were grown in presence of Ciprofloxacin (0.02-0.000625 or 8-0.25 µgmL^-1^) for 24 h in biofilm inducing conditions (1.2% bile salt). The inhibition of biofilm formation was quantified by measuring the O.D at 540nm of the crystal violet stained biofilms and graphically represented as % Biofilm formation (**a-b**) and viability was assessed by plate count method and represented as CFU mL^-1^ (**c-d**). Data are representative of three independent biological replicates and represented as +S.E.M. Statistical significance was evaluated by performing One-way ANOVA, where results are statistically significant at *p* <0.05. Group differences are marked by different superscript letters.


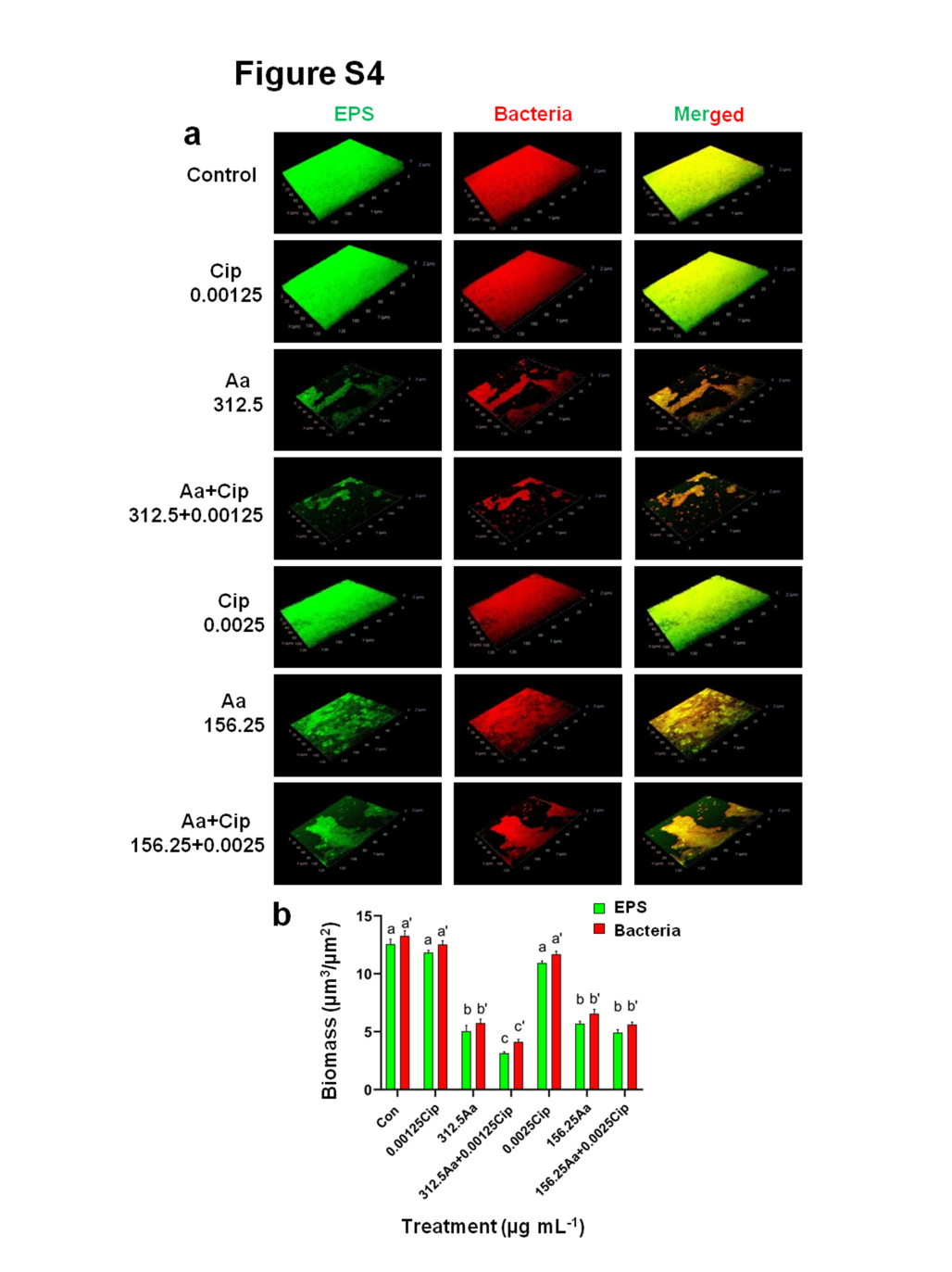


**Supplementary Figure S4. Quantification of exopolysaccharide and bacterial content of *S. flexneri* biofilm**. *S. flexneri* (Sf2457T) biofilm were grown over coverslips in presence of Asiatic acid (156.25 or 312.5µg mL^-1^) and Ciprofloxacin (0.00125 or 0.0025 µg mL^-1^) individually or in combination [(156.25Aa+0.0025Cip) or (312.5Aa+0.00125Cip)] for 4 h in biofilm inducing conditions (1.2% bile salt). Biofilms were stained with FITC conjugated Concanavalin A (green) and DAPI (pseudo red) and Z stack images of biofilms were visualized using CLSM **(a)** and biomass was quantified **(b).** Scale: 10µm. All images are representative of three independent biological replicates and data as mean +S.E.M. Statistical significance was determined using ANOVA test (two-way), where results are statistically significant at *p* <0.05. Group differences are marked by different superscript letters.


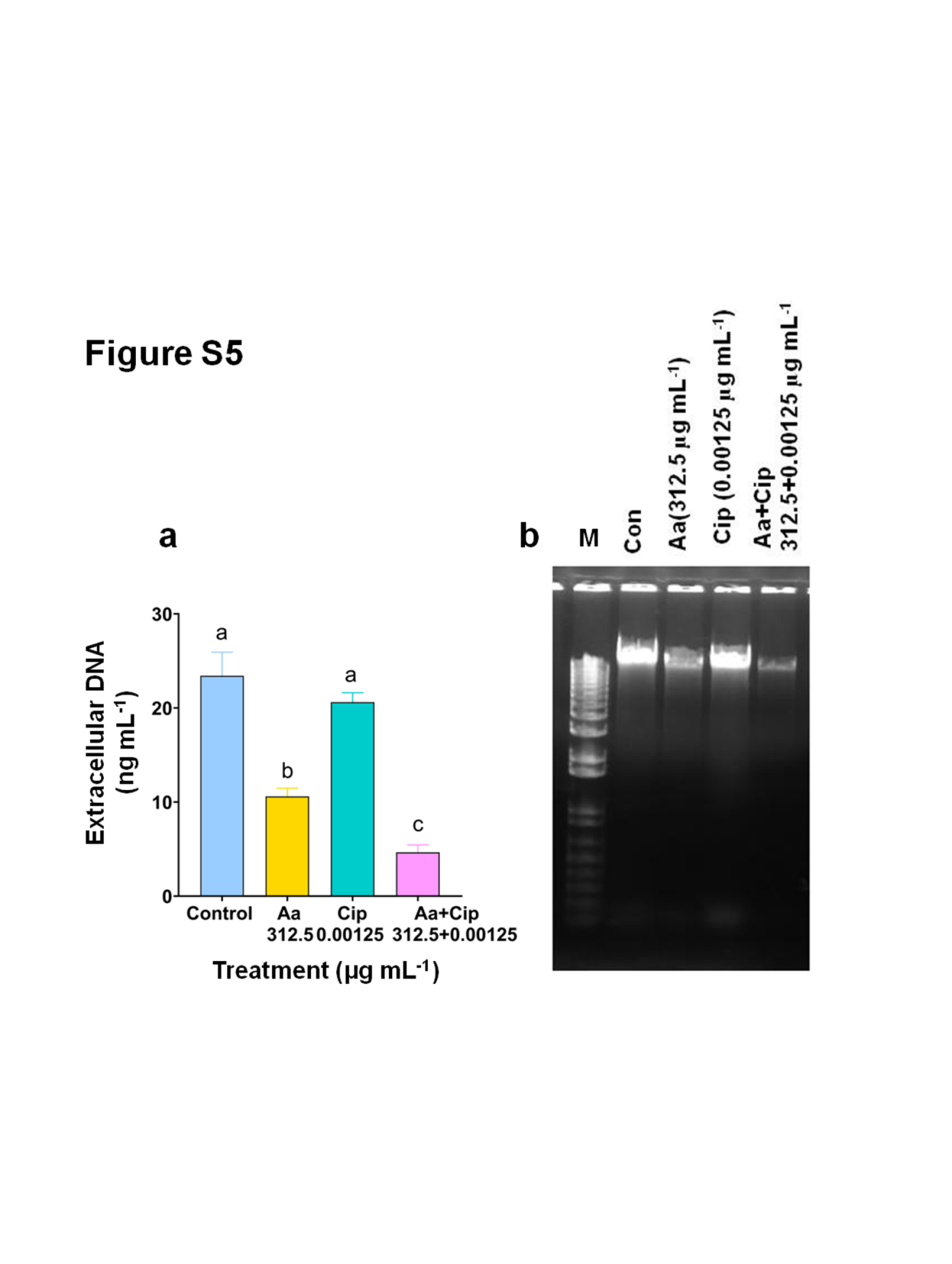


**Supplementary Figure S5. Visualization of extracellular DNA (eDNA) content of *S. flexneri* biofilm matrix**. *S. flexneri* (Sf2457T) cultures were grown in presence of Asiatic acid (312.5µg mL^-1^) and Ciprofloxacin (0.00125 µg mL^-1^) individually or in combination (312.5Aa+0.00125Cip) for 24 h in biofilm inducing conditions (1.2% bile salt). EPS was extracted using 1.5N NaCl. The eDNA content of EPS was measured at 260nm **(a)** and concentrated eDNA was visualized on Agarose gel **(b).** All images are representative of three independent biological replicates and data as mean +S.E.M. Statistical significance was determined using ANOVA test (one-way). Results are statistically significant at *p* <0.05. Group differences are marked by distinct superscript letters.


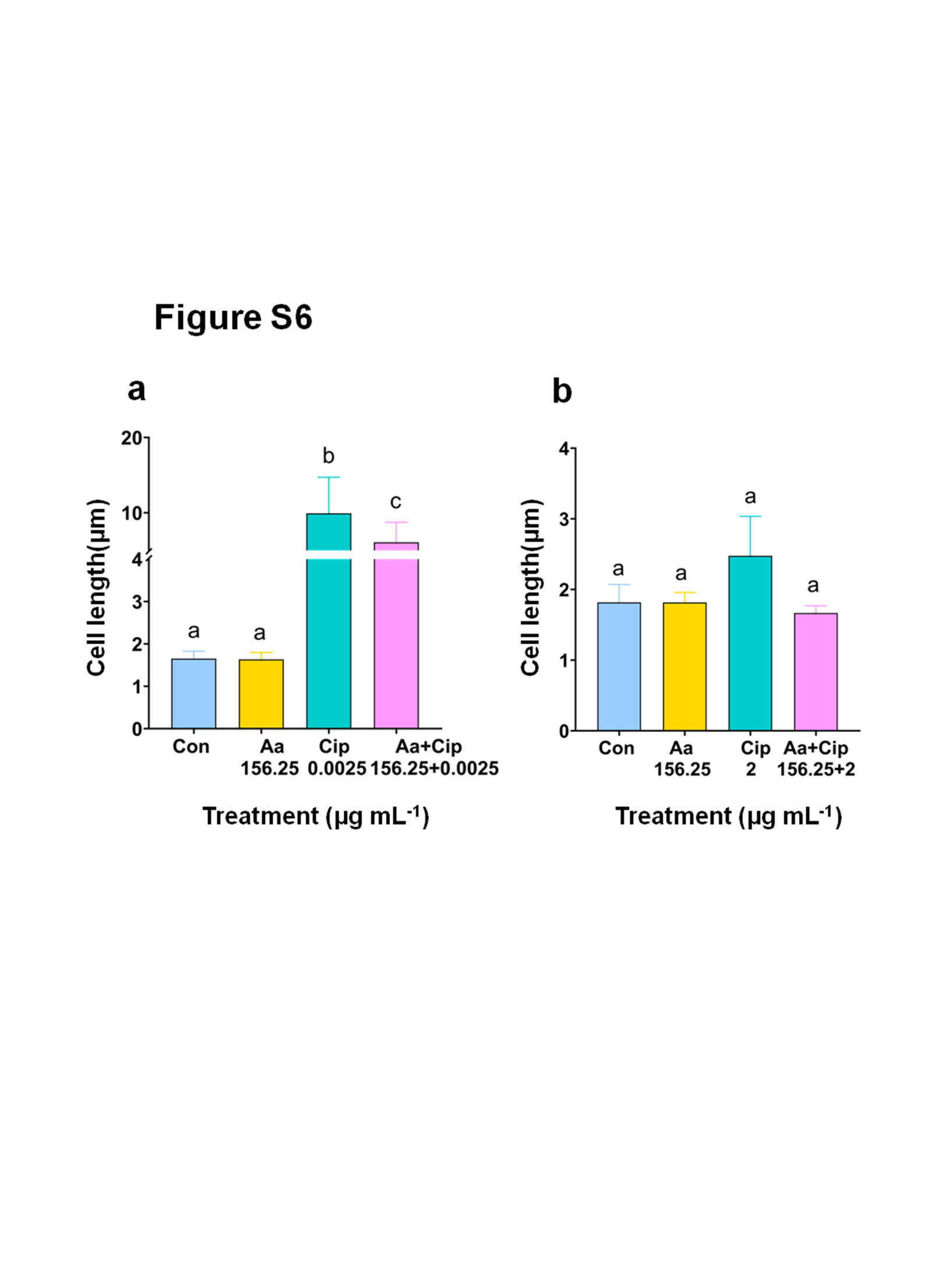


**Supplementary Figure S6. Alterations in cell length under Ciprofloxacin and Asiatic acid treatment.** *S. flexneri* (Sf2457T/ BCH12654) cultures were grown in the presence of Ciprofloxacin, Asiatic acid or in combination of both. Cells stained with Syto9/PI and visualized using CLSM was used to determine cell length in µm using ImageJ software for Sf2457T **(a)** and BCH12654 **(b)**. Data are representative of the average of 100 bacteria analyzed per group and represented as +S.E.M. Statistical significance was evaluated by performing one way ANOVA,where *p* <0.05 was considered significant. Group differences are marked by different superscript letters.


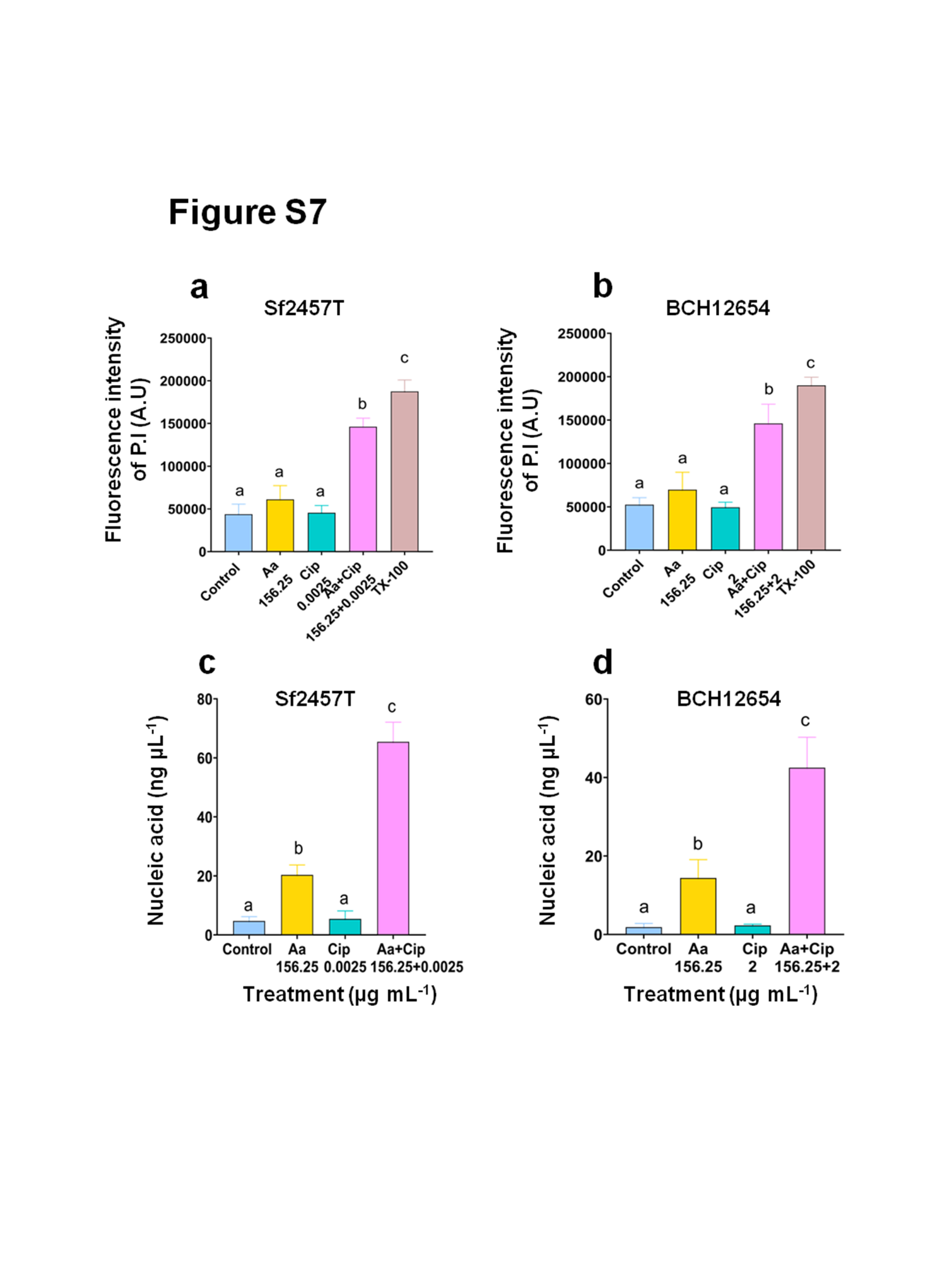


**Supplementary Figure S7. Assessment of bacterial membrane damage upon co-treatment.** *S. flexneri* (Sf2457T/ BCH12654) cultures were grown in presence of Asiatic acid, Ciprofloxacin or combination and checked for membrane integrity. Fluorescence intensity of PI-stained *S. flexneri* cultures were measured by spectrofluorometer (535/615 nm) and plotted **(a, b)**. Nucleic acid released in culture supernatant was measured using UV spectrophotometer and graphically represented as leakage in ng µL^-1^ **(c, d).** Data are representative of three independent biological replicates and represented as +S.E.M. Statistical significance was evaluated by performing one way ANOVA. Significant was considered at *p* <0.05. Differences among groups are marked by different superscript letters.


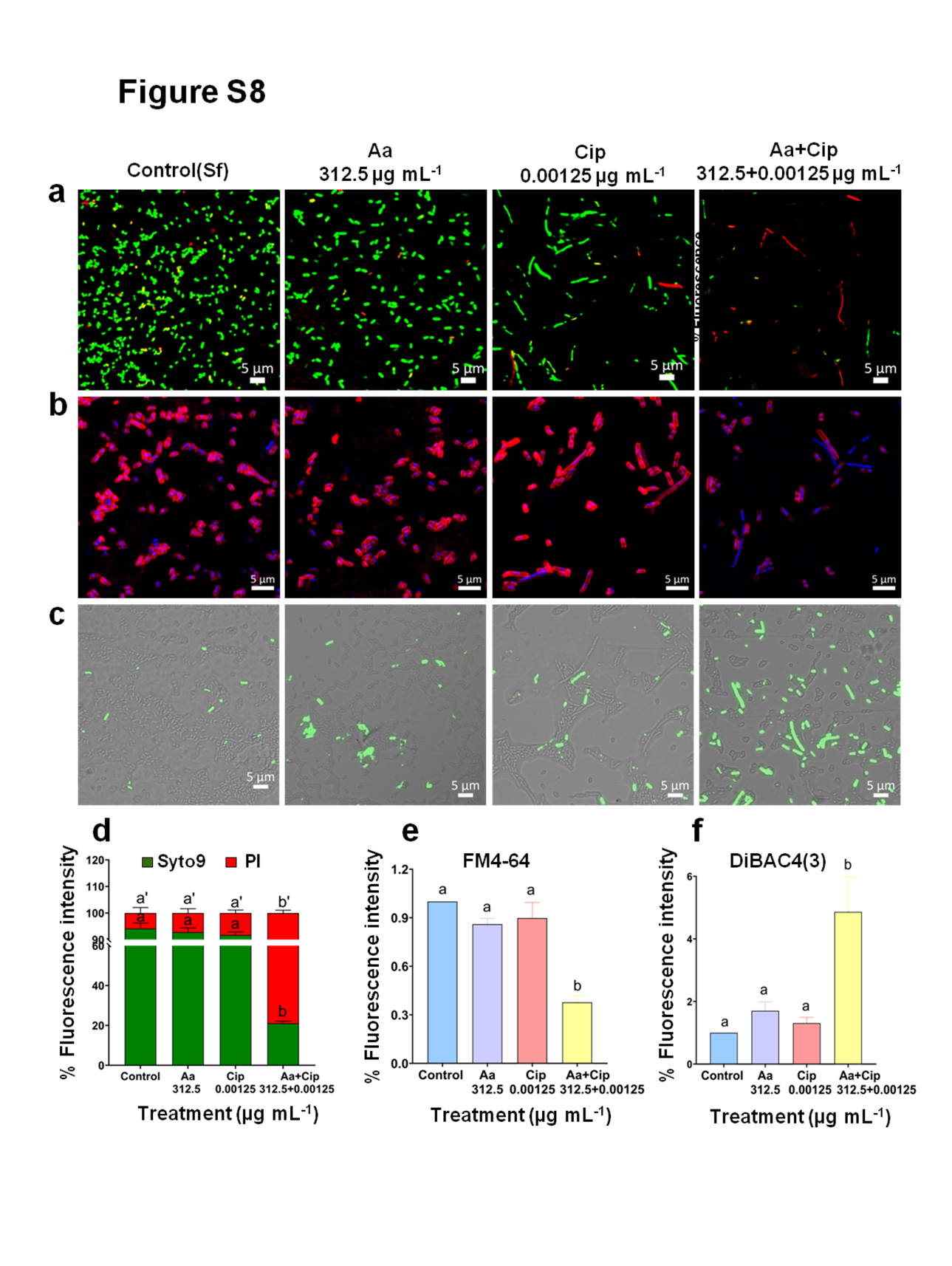


**Supplementary Figure S8. Mechanism of synergy between Asiatic acid (Aa) and Ciprofloxacin (Cip) against *S. flexneri* at a higher FICI score (0.375).** *S. flexneri* (Sf2457T) cultures were treated with mono or co-therapy of Asiatic acid (312.5 µg mL^-1^) and Ciprofloxacin (0.00125µg mL^-1^) **(a-f)**. Confocal Microscopy was used to visualize and quantify the viability of *S. flexneri* by Syto9/PI staining **(a, d)**. Membrane integrity was assessed by FM4-64 staining **(b, e)** and membrane polarity by DiBAC4 **(c, f)**. All images are representative of three independent biological replicates and data are represented as mean +S.E.M. Statistical significance was determined using ANOVA test (one or two way). Statistical significance was considered at *p* <0.05,and group differences are marked by distinct superscript letters.
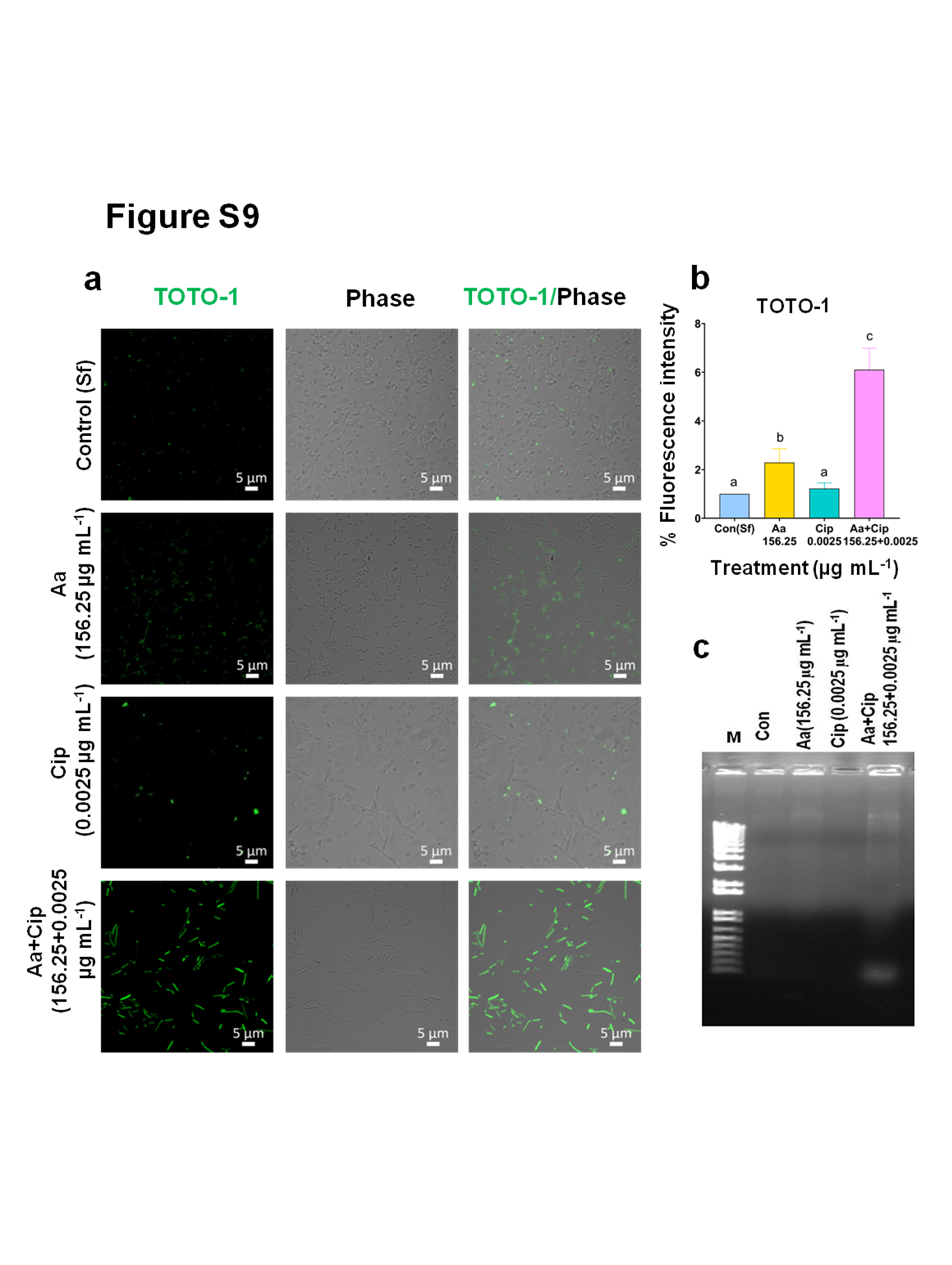


**Supplementary Figure S9. Release of nuclear material (DNA) by planktonic *S. flexneri* upon co-treatment.** *S. flexneri* (Sf2457T) cultures were treated with mono or co-therapy of Asiatic acid (156.25 µg mL^-1^) and Ciprofloxacin (0.0025 µg mL^-1^). Confocal Microscopy was used to visualize the presence of eDNA by *S. flexneri* using TOTO-1 staining **(a)** and quantified **(b)**. Nucleic acid released by treated cells was visualized by Agarose gel electrophoresis after ethanol precipitation of culture supernatant **(c)**. All images are representative of three independent biological replicates and data are represented as mean +S.E.M. Statistical significance was determined using ANOVA test (one way). *p* <0.05 was statistically significant. Differences among groups are marked by distinct superscript letters.


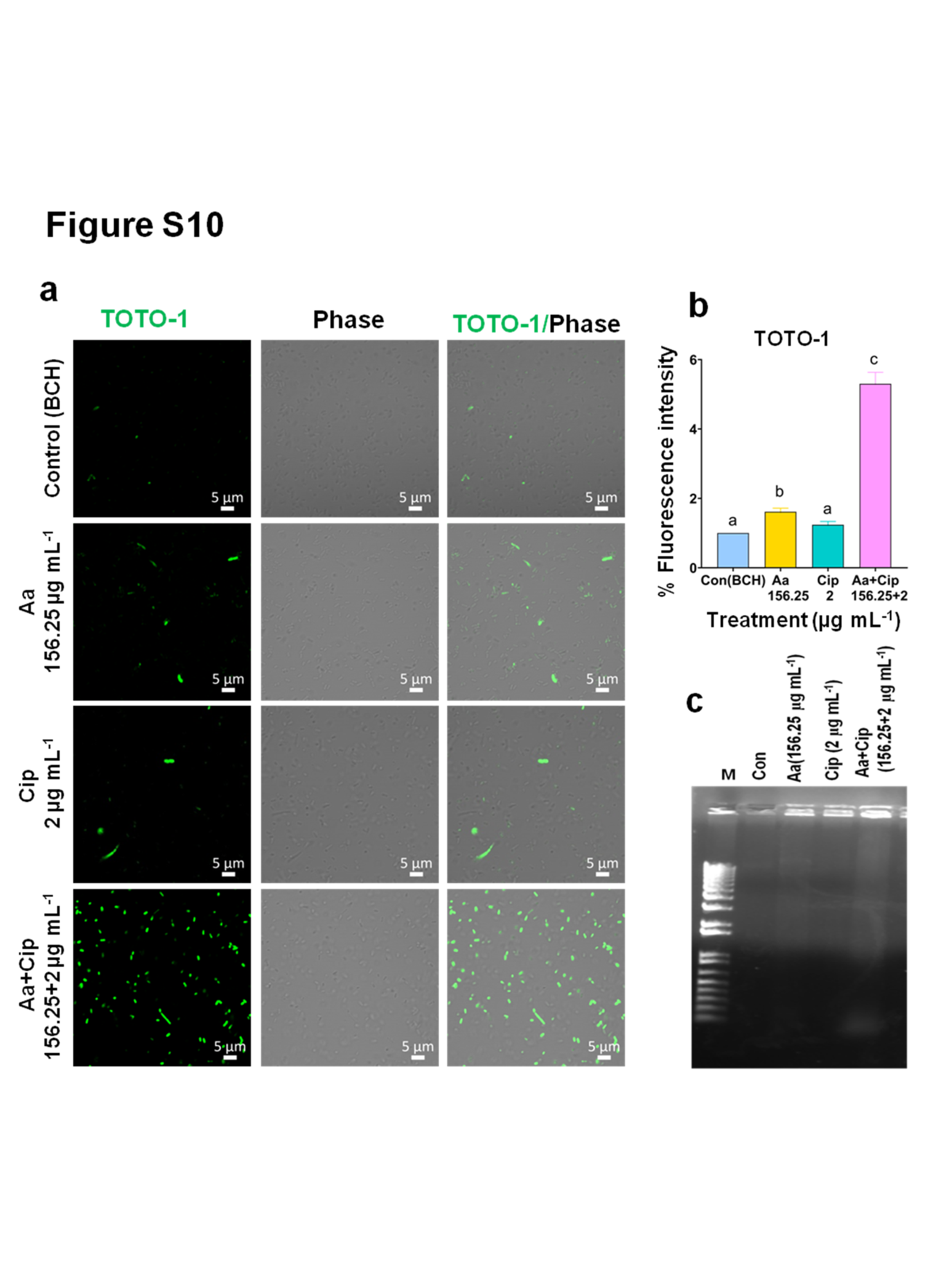


**Supplementary Figure S10. Co-treatment increased nuclear release by the resistant strain.** *S. flexneri* (BCH12654) cultures were treated with mono or co-therapy of Asiatic acid (156.25µg mL^-1^) and Ciprofloxacin (2 µg mL^-1^). Confocal Microscopy was used to visualize the release of DNA by the resistant strain using TOTO-1 staining **(a)** and quantified **(b)**. Nucleic acid released by treated cells was visualized by Agarose gel electrophoresis after ethanol precipitation of culture supernatant **(c)**. All images are representative of three independent biological replicates and data are represented as mean +S.E.M. Statistical significance was determined using ANOVA test (one way). *p* <0.05 was statistically significant and differences are marked by distinct superscript letters.


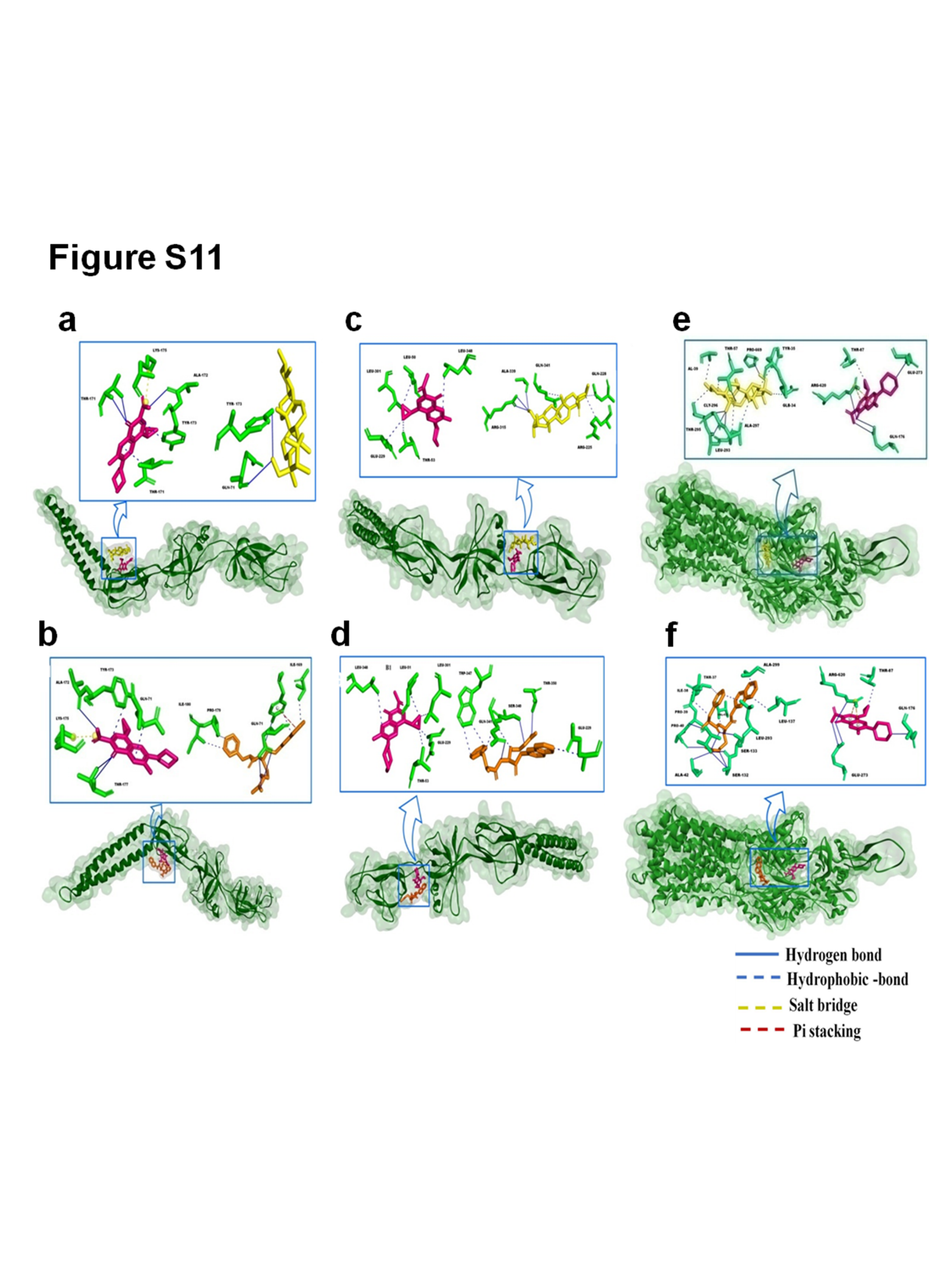


**Supplementary Figure S11. Molecular docking of Asiatic acid (Aa) and Ciprofloxacin (Cip) with AcrA/B protein.** Predicted docking positions of Asiatic acid or PAβN on Ciprofloxacin bound AcrA at the hinge site (**a, b**) and MP site (**c, d**). Docking positions of Asiatic acid or PAβN on Ciprofloxacin bound AcrB (**e, f**). The substrate Ciprofloxacin is shown in pink, ligands Asiatic acid and PAβN in yellow and orange respectively.

**Supplementary Table S3. The free energy of interaction (ΔG _binding_) calculated from the docking experiment**

| **Protein** | **Site** | **Docked ligand** | **Energy (kcal Mol^-1^)** |
| --- | --- | --- | --- |
| AcrA | Hinge | Asiatic acid | -3 |
|  |  | PaβN | -5.4 |
|  | MP | Asiatic acid | -7 |
|  |  | PaβN | -7.2 |
| AcrB | - | Asiatic acid | -8.3 |
|  | - | PaβN | -9 |

| **Gene** | **Forward Primer** | **Reverse Primer** |
| --- | --- | --- |
| 16s rRNA | TTGACGGGGGCCCGCACAAGC | AGTTCCCGGCCGGACCGCTGG |
| AcrA | ACGCGGCGTACGGGTTACGCC | AGTTCCCGCAGGACGGTACGCTGG |
| AcrB | TGCGTTCTGCGCGCCGGAACC | GCCACGCTTGATGCGGTGCGG |

**Supplementary Table S4. List of primers used for Real Time PCR**


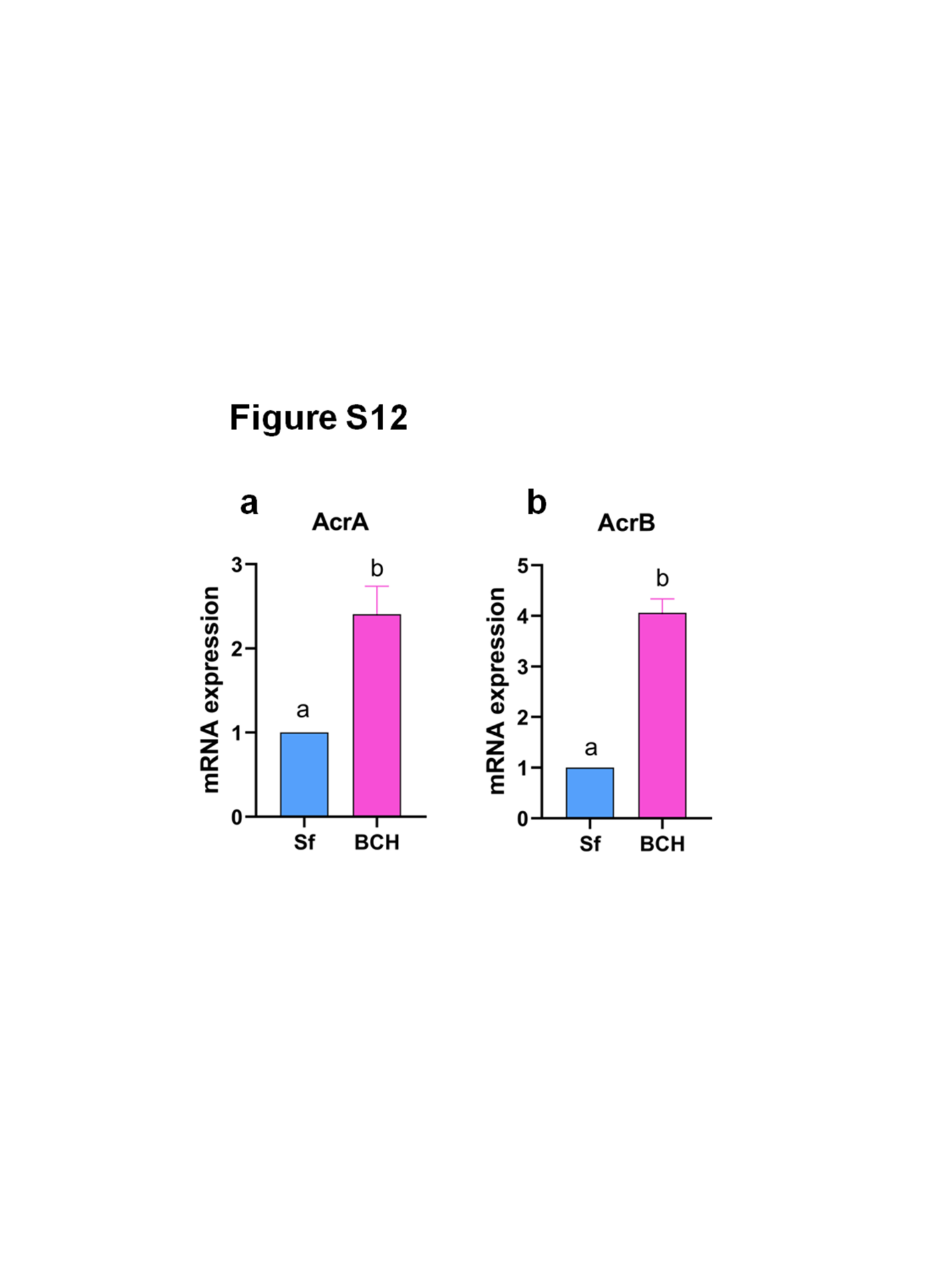


**Supplementary Figure S12. Relative expression of acrA/B genes among *S. flexneri*.** Quantitative analysis of efflux pump gene transcripts (acrA and acrB) among standard and resistant bacterial strain was performed using qRT-PCR. Expression levels were normalized to 16s rRNA. Data are representative of three independent biological replicates and represented as +S.E.M. Statistical significance was evaluated by performing t-test. Statistical significance was considered at *p* <0.05. Differences among group are marked by different superscript letters.


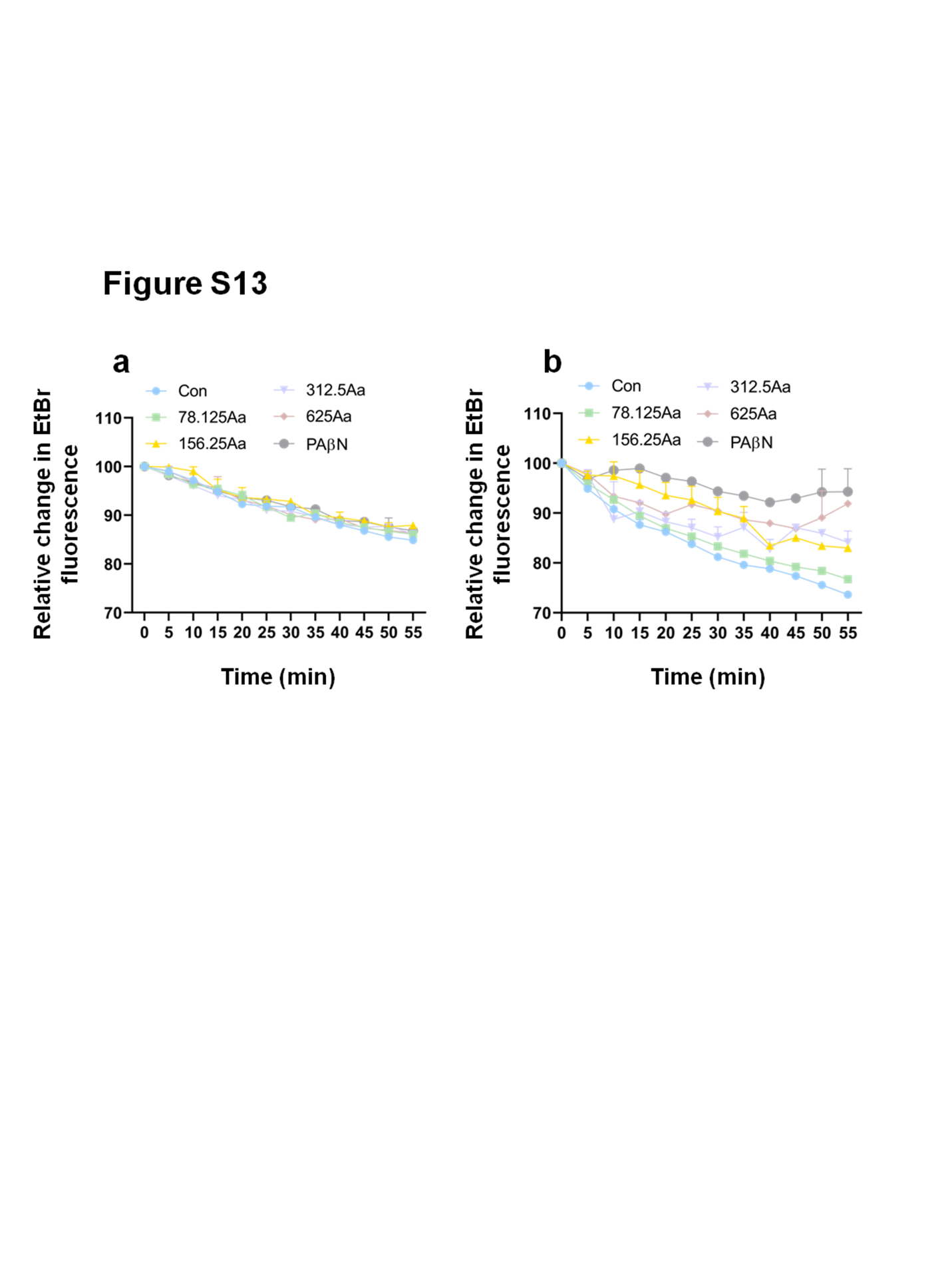
**Supplementary Figure S13. Efflux activity of *S. flexneri* in presence of Asiatic acid.** Exponential (0.6) *S. flexneri* (Sf2457T/ BCH12654) cultures were incubated with 20 µg mL^-1^ of EtBr for 1h at RT. Bacteria was then washed in PBS, adjusted to 0.2 O.D and treated with Asiatic acid and PAβN in presence of 0.4% Glucose. Change in fluorescence of EtBr was monitored over 1h. Efflux activity is calculated as relative change in fluorescence of EtBr **(a, b)**. Data are representative of three independent biological replicates and represented as +S.E.M.


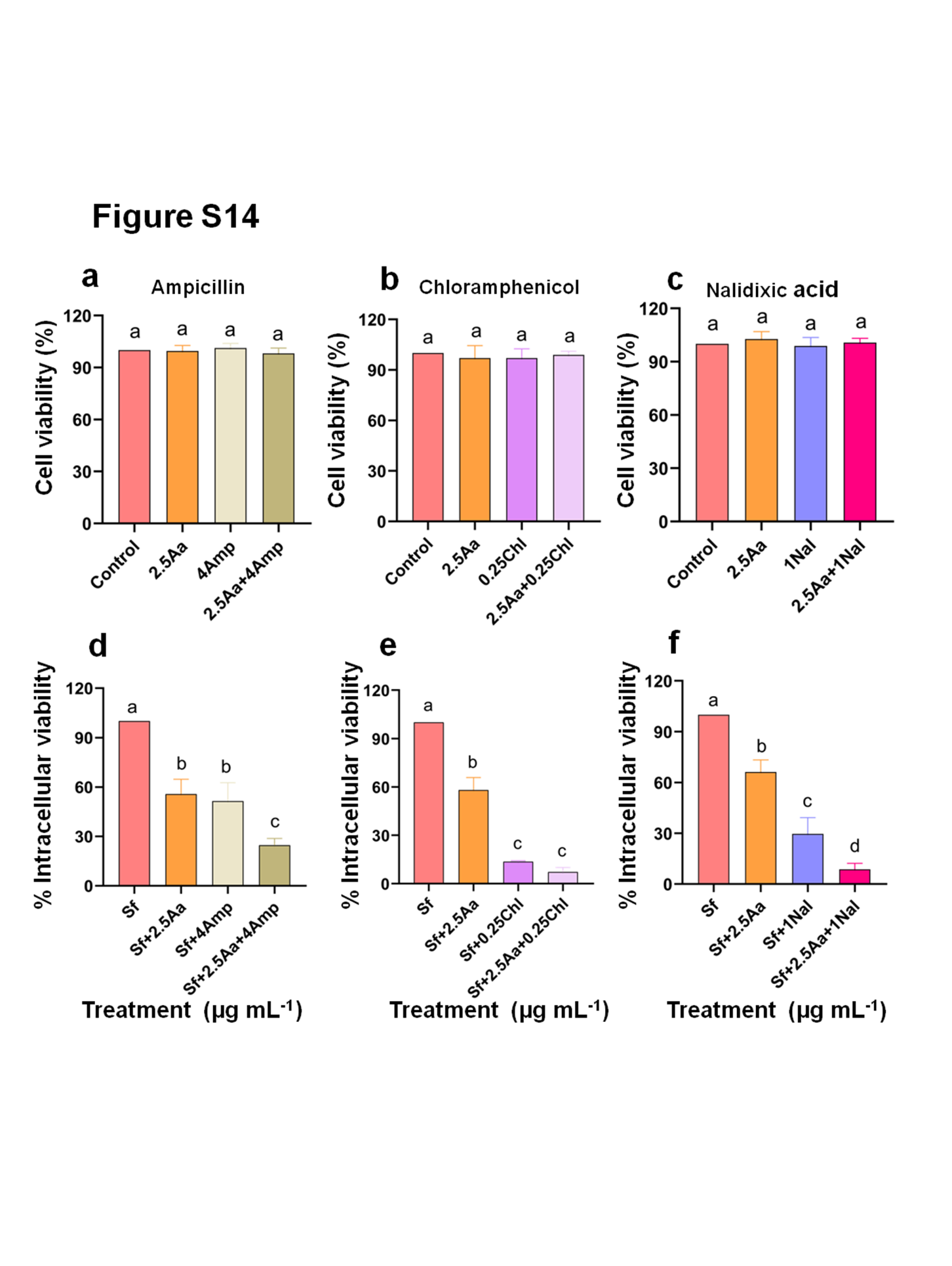


**Supplementary Figure S14. Effect of Asiatic acid (Aa) and antibiotic co-treatment on mammalian cell viability and intracellular *S. flexneri* (ATCC) growth.** HT-29 cells were treated with Asiatic acid and antibiotics (Ampicillin, Chloramphenicol and Nalidixic acid) individually and in combination for 24h, and cellular viability was assessed using MTT reagent and graphically represented as % cell viability **(a-c)**. HT-29 cells infected with *S. flexneri* (MOI 100) were treated with Asiatic acid in presence or absence of antibiotics for 24h, and the intracellular bacterial load was quantified by plate count method and graphically represented as % intracellular viability of bacteria (**d-f**). Data are representative of three independent biological replicates and represented as +S.E.M. Statistical significance was evaluated by performing one way ANOVA. *p* <0.05 was taken as statistically significant and marked by different superscript letters.
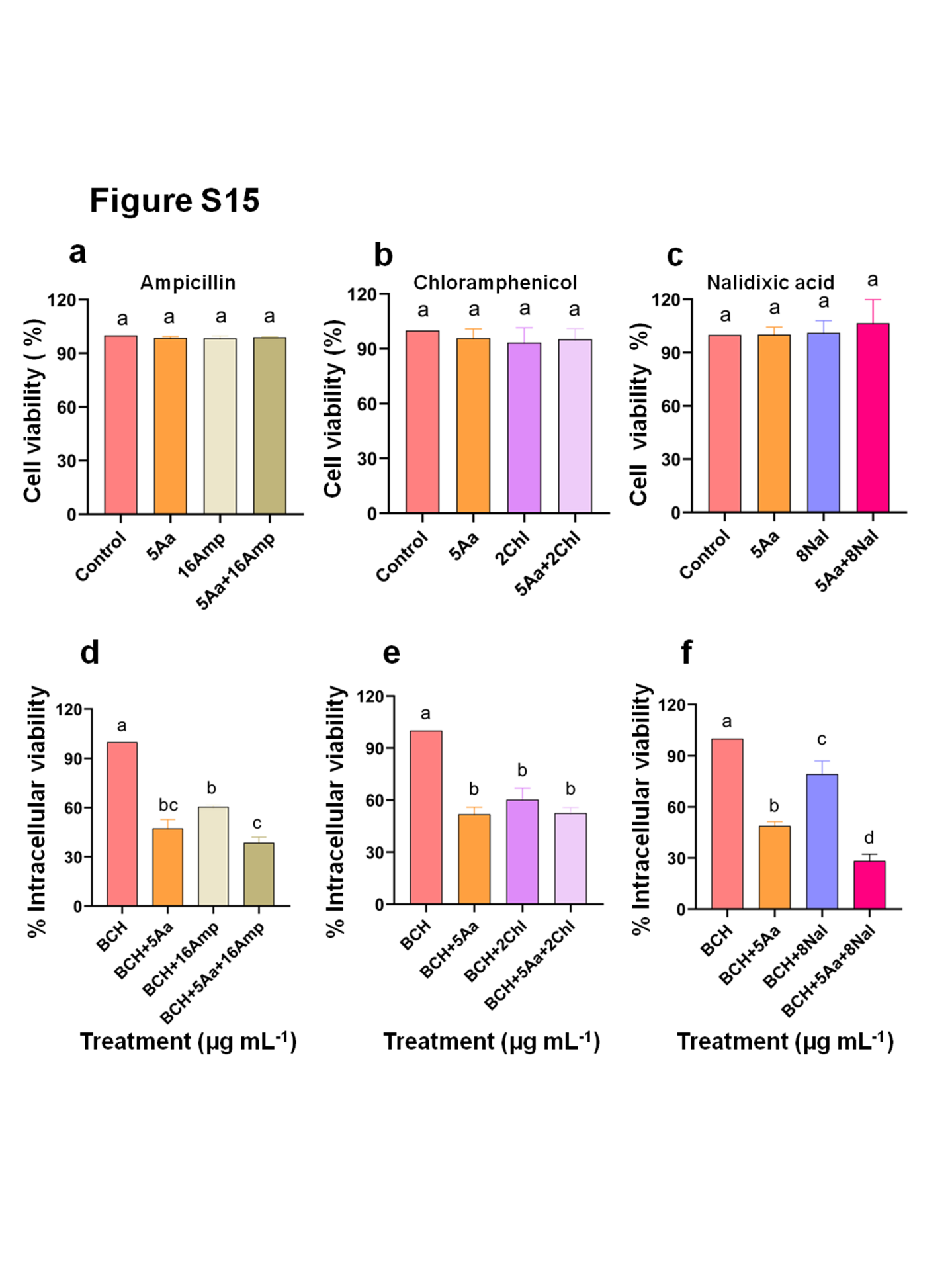


**Supplementary Figure S15. Asiatic acid (Aa) and antibiotic co-administration has differential effect on mammalian cell viability and intracellular *S. flexneri* resistant strain BCH12654 growth.** HT-29 cells were treated with mono or combination of Asiatic acid and antibiotics (Ampicillin, Chloramphenicol and Nalidixic acid) for 24h, and cellular viability was assessed using MTT reagent and graphically represented as % cell viability **(a-c)**. HT29 cells infected with *S. flexneri* (MOI 100) were treated with Asiatic acid in presence or absence of antibiotics for 24h, and the intracellular bacterial load was quantified by plate count method and graphically represented as % intracellular viability of bacteria (**d-f**). Data are representative of three independent biological replicates and represented as +S.E.M. Statistical significance was evaluated by performing one way ANOVA. *p* <0.05 was statistically significant and differences are marked by distinct superscript letters.


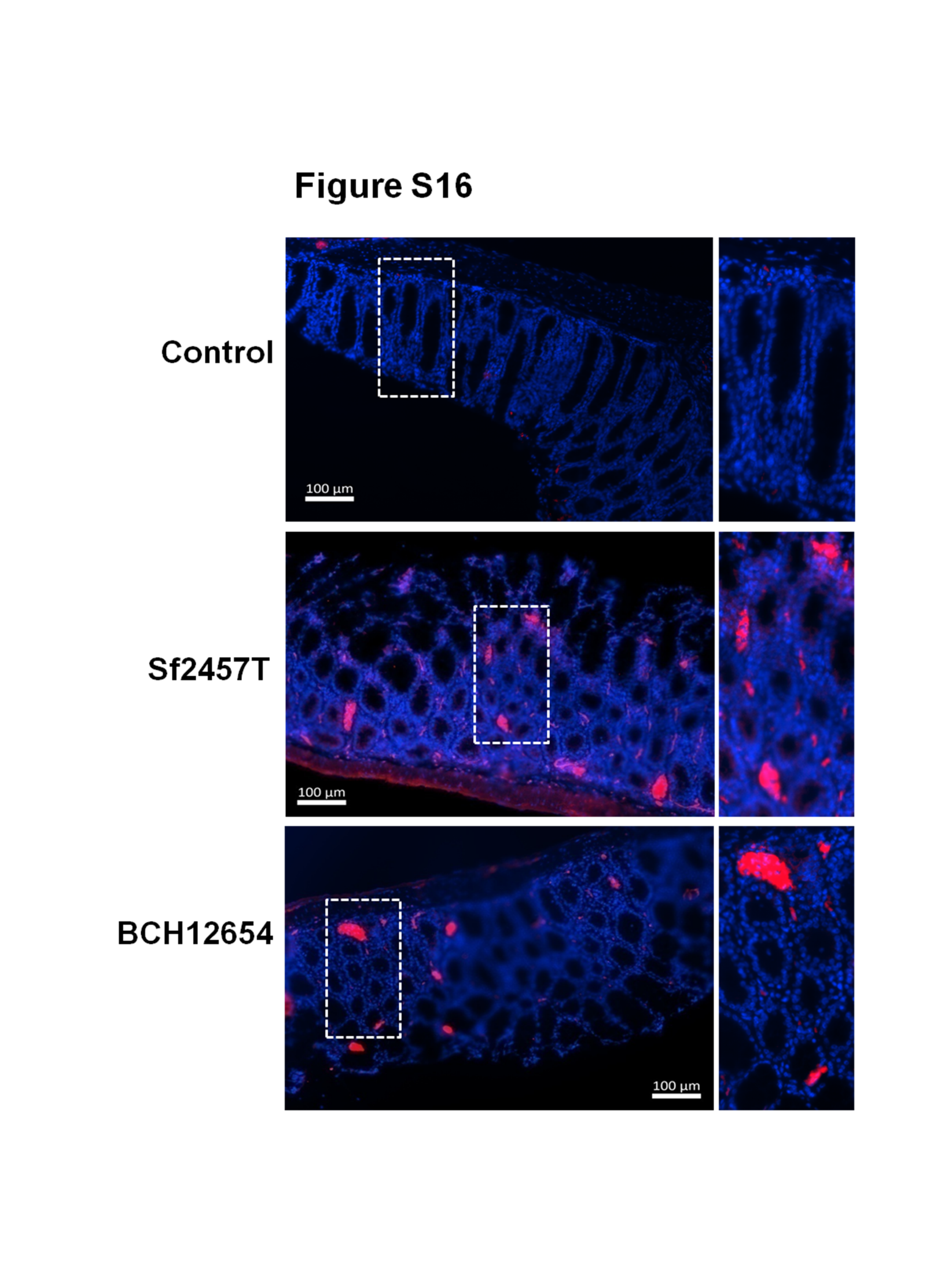


**Supplementary Figure S16. Visualization of *S. flexneri* in colon of infected mice.** Representative immunofluorescence image of colonic sections of uninfected and *S. flexneri*(Sf2457Tand BCH12654) infected mice.*S. flexneri*was stained with anti-*Shigella* antibody (red) and nucleus with DAPI (blue). Scale bar: 100 µm.


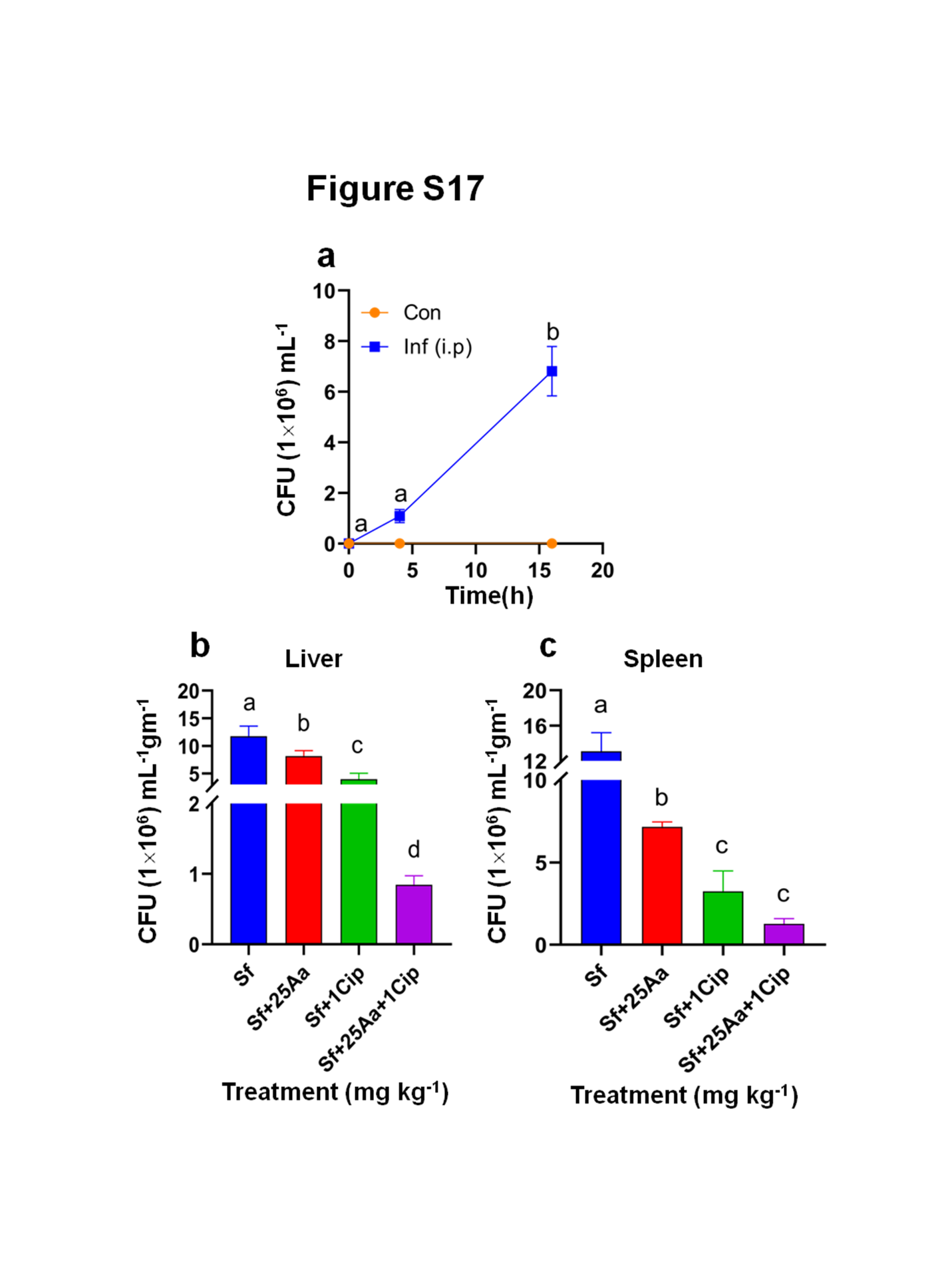


**Supplementary Figure S17. *S. flexneri* (Sf2457T) administered through intraperitoneal route causes bacteremia and infects systemic tissues.** Fasted BALB/c mice were intraperitoneally infected with *S. flexneri* (10^8^CFU), followed by treatment with Ciprofloxacin in presence or absence of Asiatic acid. Blood was drawn at 4 and 16 h and plated for CFU count **(a)**. Post treatment, bacterial load in gentamicin treated liver **(b)** and spleen **(c)** was counted by plate count method and graphically represented as CFU gm^-1^ of tissue. Data are representative of three independent biological replicates and represented as +S.E.M. Statistical significance was evaluated by performing one way ANOVA. *p* <0.05 was statistically significant and differences are marked by distinct superscript letters.
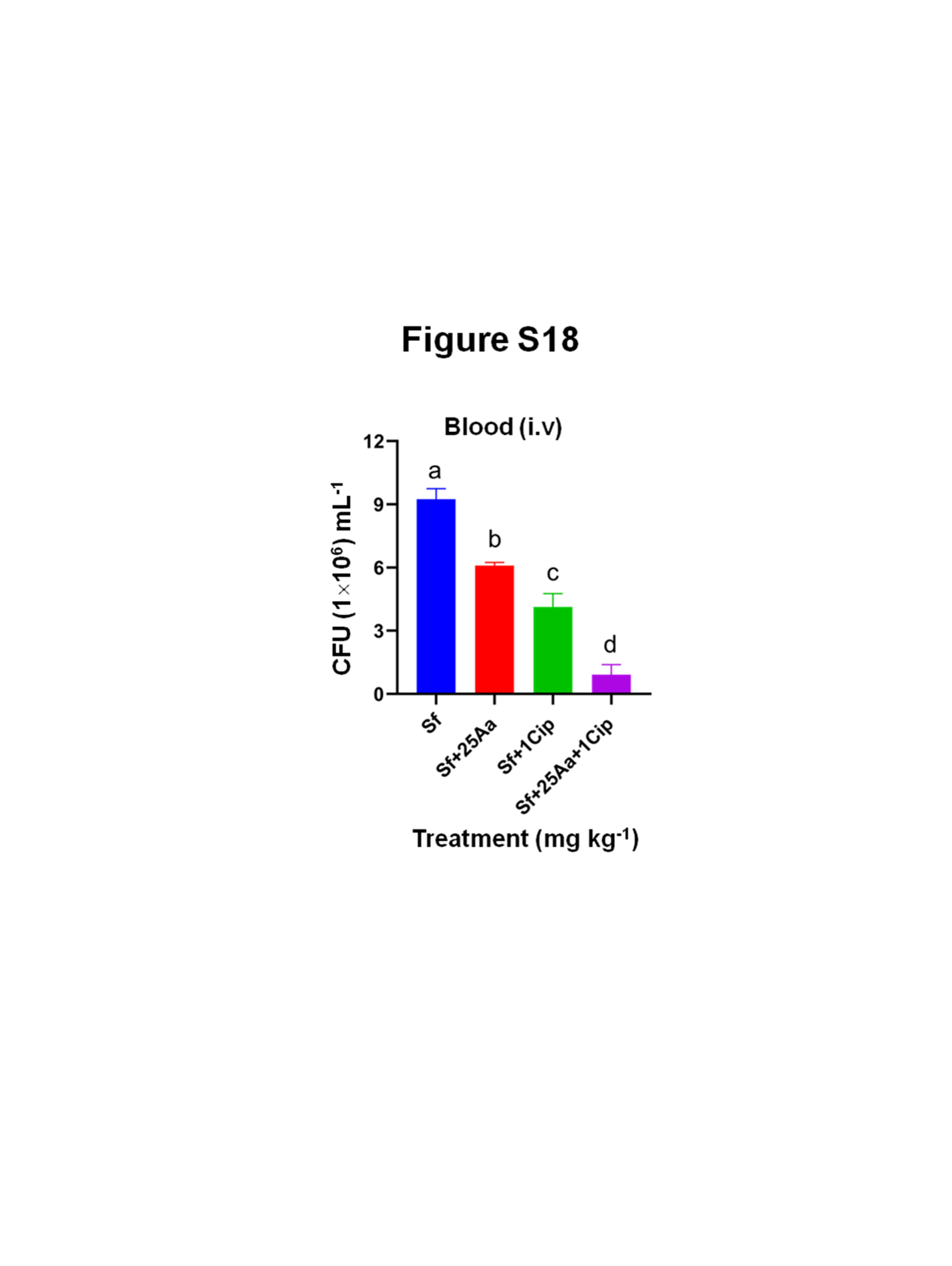


**Supplementary Figure S18. Intravenously administered *S. flexneri* in blood is lowered by co-treatment.** Fasted BALB/c mice were intravenously infected with *S. flexneri* (10^8^ CFU), followed by treatment with Ciprofloxacin in presence or absence of Asiatic acid. Post treatment, CFU count in blood was assessed by plate count method and graphically represented. Data are representative of three independent biological replicates and represented as +S.E.M. Statistical significance was evaluated by performing one-way ANOVA. *p* <0.05 was statistically significant and differences are marked by distinct superscript letters.


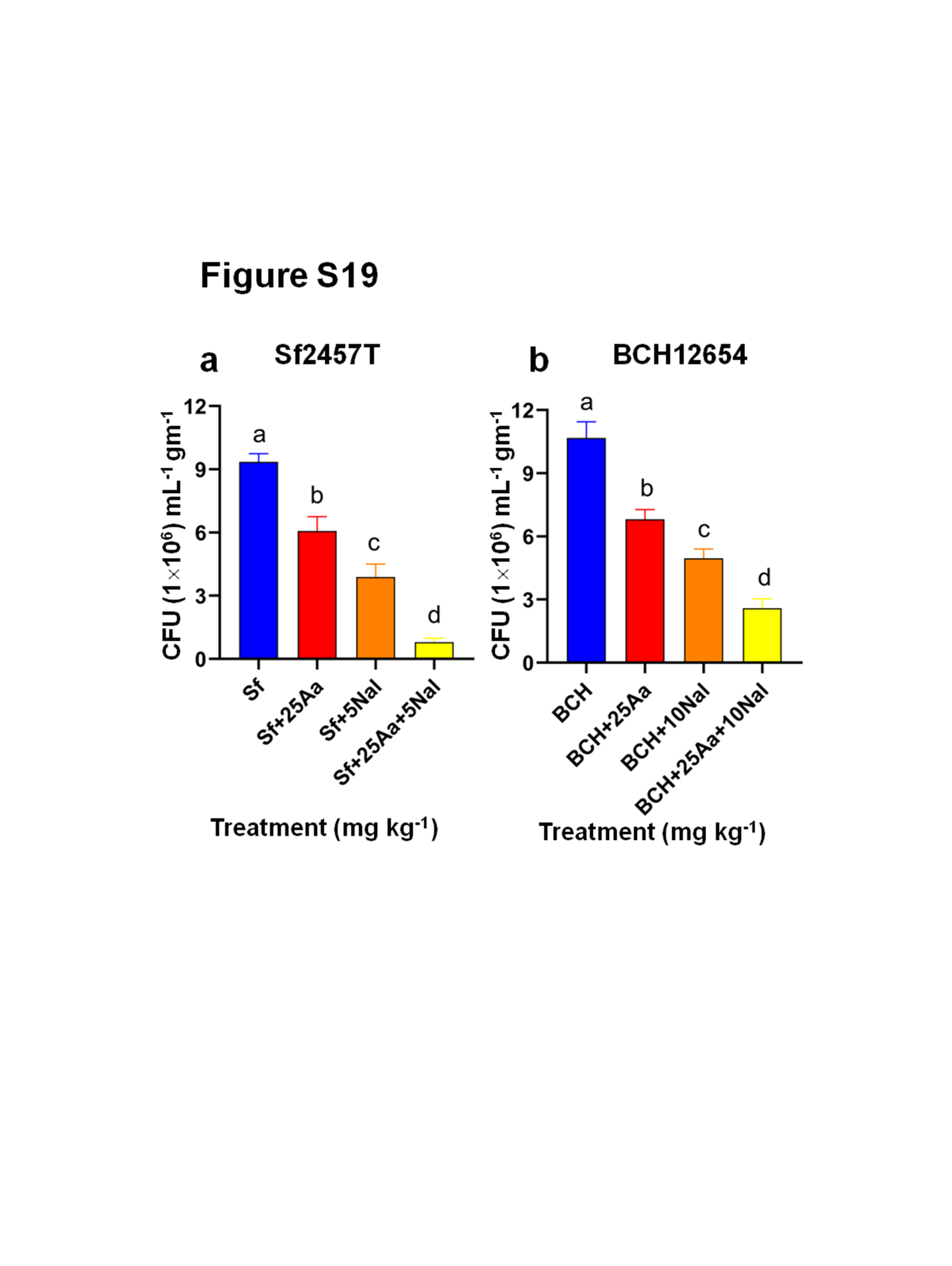


**Supplementary Figure S19. Asiatic acid (Aa) and Nalidixic acid (Nal) combination enhances *in-vivo* antimicrobial efficacy against *S. flexneri***. Fasted BALB/c mice were intraperitoneally infected with *S. flexneri* (10^8^CFU), followed by treatment with Nalidixic acid in presence or absence of Asiatic acid. Post treatment, mice were sacrificed and bacterial load in gentamicin treated colon was counted by plate count method and graphically represented as CFU gm^-1^of tissue (**a, b**). Data are representative of three independent biological replicates and represented as +S.E.M. Statistical significance was evaluated by performing one way ANOVA. *p* <0.05 was statistically significant and differences are marked by different superscript letters.


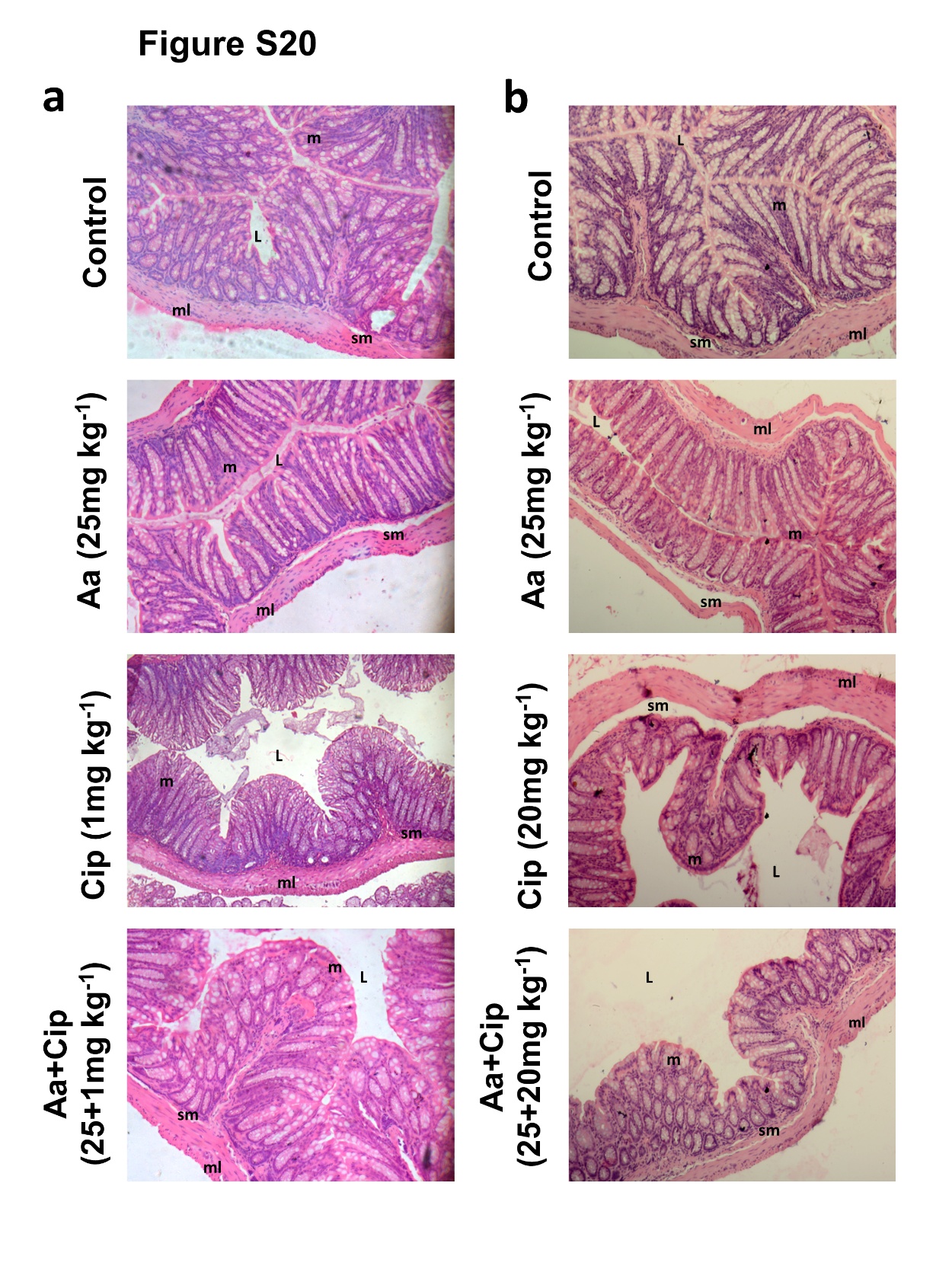


**Supplementary Figure S20. Asiatic acid (Aa) and Ciprofloxacin (Cip) administration produced no effect on un-infected mice.** Fasted un-infected BALB/c mice were orally administered with Ciprofloxacin in presence or absence of Asiatic acid. Dosage administered were **(a)** Control, Aa- 25 mg Kg^-1^, Cip- 1mg Kg^-1^, Aa+Cip- 25+1mg Kg^-1^and **(b)** Control, Aa- 25 mg Kg^-1^, Cip- 20 mg Kg^-1^, Aa+Cip- 25+20 mg Kg^-1^. After 24 h of treatment, mice were sacrificed and histopathological changes of colon were assessed by H & E staining and observed under microscope at 20X magnification. Colonic regions are labeled as; L, lumen; sm, submucosa; m, mucosa; ml, muscularis.
